# The impact of abstinence from chronic alcohol consumption on the mouse striatal proteome: sex and subregion-specific differences

**DOI:** 10.3389/fphar.2024.1405446

**Published:** 2024-06-03

**Authors:** Brittnie-lee M. Duffus, David L. Haggerty, Emma H. Doud, Amber L. Mosley, Bryan K. Yamamoto, Brady K. Atwood

**Affiliations:** ^1^ Department of Pharmacology and Toxicology, Indiana University School of Medicine, Indianapolis, IN, United States; ^2^ Department of Biochemistry and Molecular Biology, Indiana University School of Medicine, Indianapolis, IN, United States; ^3^ Stark Neurosciences Research Institute, Indiana University School of Medicine, Indianapolis, IN, United States

**Keywords:** alcohol, alcohol abstinence, chronic alcohol consumption, striatum, inflammation, proteomics

## Abstract

Alcohol misuse is the third leading preventable cause of death in the world. The World Health Organization currently estimates that 1 in 20 deaths are directly alcohol related. One of the ways in which consuming excessive levels of alcohol can both directly and indirectly affect human mortality and morbidity, is through chronic inflammation. Recently, studies have suggested a link between increased alcohol use and the incidence of neuroinflammatory-related diseases. However, the mechanism in which alcohol potentially influences neuroinflammatory processes is still being uncovered. We implemented an unbiased proteomics exploration of alcohol-induced changes in the striatum, with a specific emphasis on proteins related to inflammation. The striatum is a brain region that is critically involved with the progression of alcohol use disorder. Using mass spectrometry following voluntary alcohol self-administration in mice, we show that distinct protein abundances and signaling pathways in different subregions of the striatum are disrupted by chronic exposure to alcohol compared to water drinking control mice. Further, in mice that were allowed to experience abstinence from alcohol compared to mice that were non-abstinent, the overall proteome and signaling pathways showed additional differences, suggesting that the responses evoked by chronic alcohol exposure are dependent on alcohol use history. To our surprise we did not find that chronic alcohol drinking or abstinence altered protein abundance or pathways associated with inflammation, but rather affected proteins and pathways associated with neurodegeneration and metabolic, cellular organization, protein translation, and molecular transport processes. These outcomes suggest that in this drinking model, alcohol-induced neuroinflammation in the striatum is not a primary outcome controlling altered neurobehavioral function, but these changes are rather mediated by altered striatal neuronal structure and cellular health.

## Introduction

Alcohol Use Disorder (AUD) is a rising public health crisis that affects over 29.5 million people ages 12 and older (SAMHSA, 2021; NIAAA, 2024). AUD is characterized by a pattern of alcohol consumption that is associated with symptoms such as increased alcohol craving and consumption, and the inability to stop drinking. This disorder is associated with several neurological effects including cognitive impairment, memory loss, and anxiety (Wilcox et al., 2014; Bloch et al., 2020; Spinola et al., 2022). Its development is complex and involves a combination of genetic, environmental, and psychological factors (McLellan et al., 2000). Currently, there are three drugs approved by the US Food and Drug Administration (FDA) to treat individuals who suffer from AUD: Disulfiram (inhibitor of aldehyde dehydrogenase to increase acetaldehyde), Naltrexone (inhibitor of opioid receptor to reduce drug-related euphoria), and Acamprosate (inhibitor of NMDA glutamate receptor) (Alcoholism, 2024). However, many individuals fail to successfully abstain after treatment, and usually relapse within a year (Maisto et al., 2003). As such, AUD treatment remains a challenge as a critical gap exists concerning the neural regulation of alcohol consumption and relapse to alcohol misuse.

Chronic alcohol consumption impairs cognition, decision-making, attention, and learning by disrupting the structure and function of various brain regions (Evert and Oscar-Berman, 1995; Topiwala et al., 2017). The striatum is one such region that is significantly involved in the reinforcement of alcohol use due its role in the addiction cycle (Koob and Le Moal, 1997; Koob and Volkow, 2010). This region’s structures and functions are classically involved in the binge/intoxication stage of the addiction cycle (Substance Abuse and Mental Health Services Administration, 2016). The nucleus accumbens (NAc), also known as the ventral striatum, is heavily involved in the initial stages of drug reward and reinforcement (Gremel and Costa, 2013). The dorsal striatum (DS) also plays a major role in action selection behaviors, such as drug seeking and habit formation. The dorsomedial striatum (DMS) and dorsolateral striatum (DLS) are subregions of the DS that mediate different forms of action selection. The DMS governs goal-directed actions while the DLS governs habitual behaviors (Balleine and O'Doherty, 2010; Corbit et al., 2012; Valentin et al., 2007). As alcohol drinking behavior progresses and becomes habitual, control can shift from the NAc and DMS to the DLS (Willuhn et al., 2012; Bell et al., 2016). Studies show that striatal regions undergo modifications to synapses in response to alcohol use including receptor changes and altered connectivity between brain regions (Sulzer, 2011; Flores-Bastias and Karahanian, 2018; Lieselot et al., 2023). While prior research has uncovered much about how neural firing across the striatum correlates with alcohol drinking, the molecular mechanisms that are altered by chronic alcohol consumption remain unclear.

One way that alcohol consumption may impact brain function is by driving pro-neuroinflammatory states (He and Crews, 2008). Chronic alcohol drinking induces neuroinflammatory responses such as microglia recruitment, proliferation and activation which results in increases in pro-inflammatory markers such as cytokines and Toll-like receptor 4 (TLR4) activation (Zou and Crews, 2005; Fernandez-Lizarbe et al., 2013; Crews and Vetreno, 2016; Marshall et al., 2016; Siemsen et al., 2021; Swartzwelder et al., 2019; Crews et al., 2023). Supporting a potential causative role for inflammation in alcohol consumption, anti-inflammatory drugs can affect alcohol drinking behavior (Blaker and Yamamoto, 2018; Macht et al., 2021; Kline and Yamamoto, 2022). Previous work from our laboratory has demonstrated that inhibition of the prostaglandin E_2_ (PGE_2_) receptor as well as cyclooxygenase-2 (COX-2) reduce alcohol reinstatement in adolescent female rats after a period of abstinence from alcohol drinking (Kline and Yamamoto, 2022). A potential molecular mechanism that underlies these effects is that alcohol drinking increased expression of the pro-inflammatory mediators, endothelin-1 (ET-1), COX-2, and PGE_2_ during alcohol abstinence which may account for the ability of the anti-inflammatory treatments to reduce reinstatement of drinking (Kline and Yamamoto, 2022). Of particular interest, these molecular changes were only observed in the DS, but not the NAc, suggesting that elevated inflammation locally in the DS may drive reinstatement of alcohol drinking after abstinence.

To follow up on this work, we performed an unbiased proteomic assessment of the effects of alcohol drinking on dorsal and ventral striatum protein expression. Our goal was to identify all potential inflammatory mediators that may contribute to alcohol drinking and alcohol reinstatement following a period of abstinence. To do so, we used a model of chronic alcohol consumption with acute and protracted alcohol abstinence in adult male and female mice followed by proteomics measures. We hypothesized that there would be differences in the abundance of proteins that are an expanded part of the same signaling pathways as was seen in our rat model.

## Methods

### Animals

All animal research was done according to approved protocols by the Indiana University School of Medicine Institutional Animal Care and Use Committee (IACUC) and with guidelines established by the National Institutes of Health (NIH). 48 seven-week-old mice (24 males and 24 females) were obtained from Jackson Laboratories (Bar Harbor, ME) and single housed in a reversed light cycle room (12h light/dark cycle). Mice were allowed to acclimatize for 1 week before commencing drinking.

### Voluntary intermittent access two-bottle choice drinking

All water and ethanol drinking were done using home cage lickometers that were constructed in-house as described in previous work done in our lab (Haggerty et al., 2022). The lickometers hold two tubes simultaneously, with one on each side. Control animals had access to two tubes of water while alcohol drinkers had access to one water tube and an alcohol tube during each drinking session. Alcohol and water tubes were weighed and placed in cages on Mondays, Wednesdays and Fridays of each week, 3 h after the light cycle ended (09:00 h). Each session lasted 24 h. At the end of each session, the bottles were immediately weighed, and water bottles were placed in the cages until the next session. Animals were given an increasing concentration of alcohol (3%, 6% and 10% v/v in water) for two sessions each, and then given a concentration of 20% for the remaining drinking sessions. Animals drank for a total of 15 sessions. Mice were weighed at the end of the final drinking session each week. Alcohol and water consumption was calculated in grams per kilograms (g/kg) by using the weight difference of the bottles and the density of alcohol and water, respectively. Alcohol preference percentage was calculated by dividing the total volume of alcohol consumed by the volume of total fluid (water plus alcohol) and multiplying by 100. One group of animals underwent 3 weeks of abstinence from alcohol drinking (protracted abstinence) and a second group underwent 24 h of abstinence from alcohol drinking (acute abstinence). Animals were given *ad libitum* access to food. Supplementary Figure S1 displays the experimental design and timeline. Supplementary Figure S1 was made using BioRender software with a license to publish it.

### Proteomics

All proteomics experiments were performed in collaboration with the Indiana University Proteomics Core similarly to previous work done in our lab (Grecco et al., 2021; Grecco et al., 2022; Haggerty et al., 2023).

### Tissue dissection

Tissue collection was done 24 h after the final drinking session for the acute abstinence cohort and 3 weeks after the final session for the protracted abstinence cohort. One female water drinker in the protracted abstinence group was excluded from tissue collection due to a homecage malfunction which resulted in the animal leaving the cage during the drinking session. Animals were rapidly decapitated after a brief exposure to anesthesia and tissue was dissected. Slices were cut in a 0.5 mm coronal mouse brain matrix, and DLS, DMS and NAc sections were dissected from each slice. Tissue was immediately snap frozen in isopentane on dry ice and stored at −80 ℃ until later processing.

### Protein preparation

We prepared proteins in the same way as our previous works (Grecco et al., 2021; Grecco et al., 2022; Haggerty et al., 2023): Flash frozen brain sections were homogenized in 150–170 µL of 8 M urea in 100 mM Tris, pH 8.5 using a Bioruptor^®^ sonication system (Diagenode Inc. United States, North America Cat No: B01020001) with 30 s/30 s on/off cycles for 20 min in a water bath at 4°C. After subsequent centrifugation at 10,000 rcf for 20 min, protein concentrations were determined by Bradford protein assay (BioRad Cat No: 5000006). Approximately 25 µg of protein from each sample were then treated with 5 mM tris(2-carboxyethyl) phosphine hydrochloride (Sigma-Aldrich Cat No: C4706) to reduce disulfide bonds and the resulting free cysteine thiols were alkylated with 10 mM chloroacetamide (Sigma Aldrich Cat No: C0267). Samples were diluted with 50 mM Tris.HCl pH 8.5 (Sigma-Aldrich Cat No: 10812846001) to a final urea concentration of 2 M for overnight Trypsin/Lys-C digestion at 35°C (1:25 protease:substrate ratio, Mass Spectrometry grade, Promega Corporation, Cat No: V5072.) (Levasseur et al., 2019; Li et al., 2020).

### Peptide purification and labeling

Digestion was halted by addition of 0.3% v/v trifluoroacetic acid (Millipore Cat No: 152166), and peptides were desalted on Waters Sep-Pak^®^ Vac cartridges (WatersTM Cat No: WAT054955) with a wash of 1 mL 0.1% TFA followed by elution in 0.6 mL of 70% acetonitrile 0.1% formic acid (FA). Peptides were dried by speed vacuum and resuspended 50 mM triethylammonium bicarbonate (TEAB; Sigma Cat No: 102614922). Each sample was labeled for 2 h at room temperature with 0.5 mg of Tandem Mass Tag Pro (TMTpro) reagent resuspended in acetonitrile (16-plex kit, manufactures instructions Thermo Fisher Scientific, TMTpro™ Isobaric Label Reagent Set; Cat No: 44520, Lot no. XK347989) (Li et al., 2020). Samples were checked for >90% labeling efficiency prior to being quenched with 0.3% hydroxylamine (v/v) at room temperature for 15 min. Labeled peptides were then mixed and dried by speed vacuum.

### Internal reference channel

Since the maximum number of samples that can be multiplexed using TMTpro is currently 18, in order to compare samples across multiple multiplexes, an internal reference channel was created for each brain region by pooling an equivalent amount of digested peptide from each sample after resuspension in TEAB, but prior to TMTpro labeling (Plubell et al., 2017). This pooled sample was labeled with TMTpro 134C, and divided equally to be mixed with each individual multiplex prior to high pH fractionation.

### Trigger channel creation

Since data dependent mass spectrometry can be stochastic in which specific peptides are selected for isolation, fragmentation and thus quantification, we created a trigger channel (Peck Justice et al., 2021) containing peptides from a selection of inflammatory proteins of interest identified by Western blot in our prior work (Blaker and Yamamoto, 2018). We selected peptides from previous mass spectrometry analysis (Grecco et al., 2021; Grecco et al., 2022; Haggerty et al., 2023), and when not previously identified by mass spectrometry, from high scoring peptides on peptideatlas.org (Desiere et al., 2006). Peptides were purchased from Biosynth, and a 100 µg of an equimolar solution of 8 peptides were labeled with TMTPro channel 135N. 1 ug of this labeled peptide mixture was added to each multiplex prior to high pH fractionation. Supplementary Table S1 shows the proteins and peptides represented in the trigger channel.

### High pH basic fractionation

For high pH basic fractionation, the combined, dried peptides for each multiplex were reconstituted in 0.1% FA and fractionated into 8 fractions using Pierce™ High pH reversed-phase peptide fractionation kit (Thermo Fisher Scientific Cat No 84868).

### Nano-LC-MS/MS analysis

Nano-LC-MS/MS analysis was performed as described previously (Grecco et al., 2021; Grecco et al., 2022; Haggerty et al., 2023) on an EASY-nLC™ HPLC system (SCR: 014993, Thermo Fisher Scientific) coupled to Orbitrap Fusion™ Eclipse™ mass spectrometer (Thermo Fisher Scientific). One fourth of each global peptide fraction was loaded onto a reversed phase 25 cm aurora column (IonOpticks, AUR2-25075C18A) at 400 nL/min in the EASY-nLC HPLC system (SCR: 014993, Thermo Fisher Scientific). The gradient used for the separation is 5%–30% with mobile phase B for 160 min, 30%–80% B over 10 min, and 80%–10% B in last 10 min [Mobile phases A: 0.1% FA, water; B: 0.1% FA, 80% Acetonitrile (Thermo Fisher Scientific Cat No: LS122500)]. Nano-LC-MS/MS data were acquired in Orbitrap Eclipse™ Tribid mass spectrometer (Thermo Fisher Scientific) installed with a FAIMS pro interface. The mass spectrometer was operated in positive ion mode with advanced peak determination and Easy IC™ on and with 3 FAIMS CVs (−45, −55, −70), 1.3 s cycle time per CV. Precursor scans (m/z 400–1600) were done with an orbitrap resolution of 120,000, RF lens% 30, maximum inject time 105 ms, standard AGC target, MS2 intensity threshold of 2.5e4, including charges of 2–6 for fragmentation with 60 s dynamic exclusion. MS2 scans were performed with a quadrupole isolation window of 0.7 m/z, 34% HCD CE, 50,000 resolution, 200% normalized AGC target, dynamic maximum IT, fixed first mass of 100 m/z. The data were recorded using Thermo Fisher Scientific Xcalibur (4.3) software (Thermo Fisher Scientific Inc.).

### Proteome data processing

We analyzed our proteomics data as we have done before (Grecco et al., 2021; Grecco et al., 2022; Haggerty et al., 2023): resulting RAW files were analyzed in Proteome Discover™ 2.5 (Thermo Fisher Scientific, RRID: SCR_014477) with a *Mus musculus* UniProt FASTA Reference proteome downloaded 022823 plus common contaminants. SEQUEST HT searches were conducted with a maximum number of 3 missed cleavages; precursor mass tolerance of 10 ppm; and a fragment mass tolerance of 0.02 Da. Protein FDR validator node was set to a strict target FDR of 0.01 and relaxed of 0.05. Resulting normalized abundance values for each biological sample, abundance ratio and log2 (abundance ratio) values; and respective *p*-values (protein abundance-based ratio calculation and ANOVA test) from Proteome Discover™ were exported to Microsoft Excel. Static modifications used for the search were 1) carbamidomethylation on cysteine (C) residues; 2) TMTpro label on lysine (K) residues. Dynamic modifications used for the search were TMTpro label on N-termini of peptides, oxidation of methionines, phosphorylation on serine, threonine or tyrosine, and acetylation, methionine loss or acetylation with methionine loss on protein N-termini. Percolator False Discovery Rate was set to a strict setting of 0.01 and a relaxed setting of 0.05. IMP-ptm-RS node was used for all modification site localization scores. Values from both unique and razor peptides were used for quantification. In the consensus workflows, peptides were normalized by total peptide amount with no scaling. Quantification methods utilized TMTpro isotopic impurity levels available from Thermo Fisher Scientific. Reporter ion quantification was allowed with S/N threshold of 7 and co-isolation threshold of 50%. Individual multiplex comparisons were analyzed using Proteome Discover™ were exports directly while comparison of multiple multiplexes was done after exporting PSM level intensity data to Excel.

### Gene ontology and biological pathways enrichment analysis

All protein abundance is displayed as the log2 abundance ratios of alcohol/water. Proteins that that had abundances with a *p*-value of less than 0.05 were normalized across animals by their UniProt Accession numbers and displayed using dendrograms. Proteins were clustered according to their distinct interactions. The related Gene IDs were loaded into g:Profiler (https://biit.cs.ut.ee/gprofiler) to determine protein-protein interaction and their associated networks. A Benjamini-Hochberg FDR significant threshold was selected and a user threshold of 0.01. Gene Ontology (GO) data was filtered for GO Biological Process. This data was then inserted into Revigo (http://revigo.irb.hr) (Supek et al., 2011), an online tool that summarizes extensive lists of GO terms by clustering like terms under main categories. The resulting list was set to medium (0.5), the associated values used were the adjusted *p*-values, and the species was set to *Mus musculus*. Biological pathways were assessed using the KEGG database. The resulting data were analyzed for proteins and pathways in the DLS, DMS and NAc that were common to the acute and protracted abstinence groups. This was done using the Venn diagram creator function in Bioinformatics and Revolutionary Genomics (https://bioinformatics.psb.ugent.be/webtools/Venn/). The full results from the GO analyses are available at: https://github.com/bduffus/Chronic-Alcohol-Abstinence-Proteome.

### Statistics and data visualization

Data are expressed as scatterplots and line graphs. The lines of drinking data graphs represent the mean while the shaded regions represent standard deviation. Box plots show the data extending from the 25th to 75th percentiles with whisker characterizing minimum and maximum values and diamonds identifying outlying data points. Heatmaps show a subgroup of significantly regulated proteins in males and females of each abstinence group. Seaborn and matplotlib python libraries were used to construct graphs, box plots and heatmaps. Statistical analyses were performed using GraphPad Prism (GraphPad Software V10.1.1) and pingouin. All analyses are based on an *a priori* cut-off α value for significance of *p* < 0.05. A two-way mixed analyses of variances (ANOVA) was used (for data with missing values due to leaks in drinking tubes) to analyze the drinking data from the voluntary two-bottle choice intermittent access drinking, with sex, and treatment (fluid) as the factors. Proteomics analyses were done by normalizing the alcohol/water abundance ratio to the group mean within each drinking group by sex.

## Results

### Females drank more water and alcohol over time for both the acute and protracted abstinence groups

**FIGURE 1:**
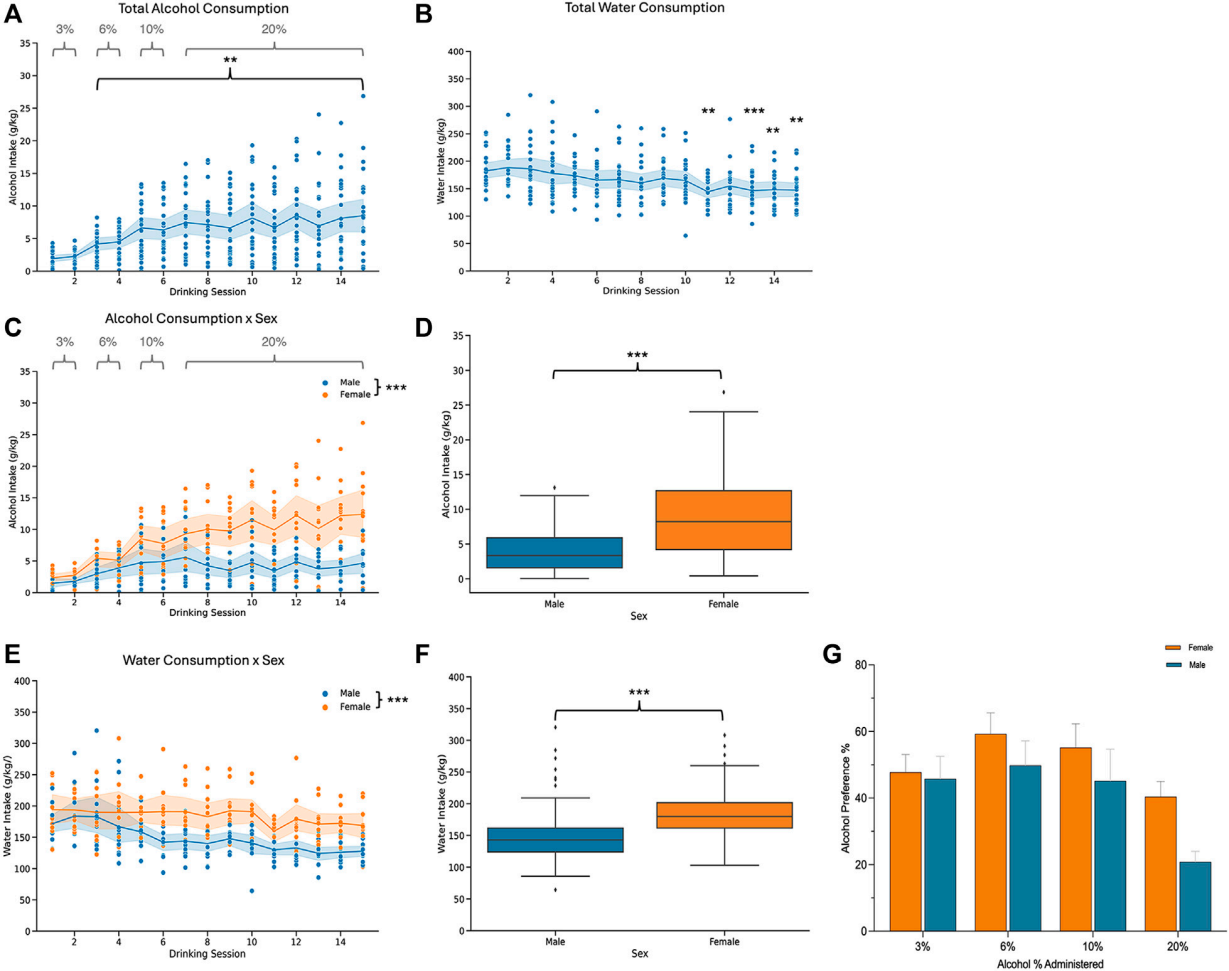
Alcohol and water consumption for male and female mice that underwent two-bottle choice intermittent voluntary drinking. **(A)** Total alcohol consumption increased overtime [*n* = 24; one-way ANOVA: *F*_(3.658, 83.08)_ = 11.06, *p <* 0.0001] and **(B)** there was a time effect on total water consumption [*n* = 23; one-way ANOVA: *F*_(3.897, 85.46)_ = 6.648, *p* < 0.0001] for all mice. **(C)** Alcohol consumption increased in males and females over time [two-way ANOVA: *F*_(4.576,99.37)_ = 12.94, *p* < 0.0001]. Females consumed a larger amount of alcohol than males [two-way ANOVA: *F*_(1,22)_ = 20.77, *p* = 0.0002] and a sex x time interaction was also found [two-way ANOVA: *F*_(14,304)_ = 4.792, *p* < 0.0001]. **(D)** Total alcohol consumption for males and females. **(E)** Time influenced water consumption [two-way ANOVA: *F*_(3.963,82.95)_ = 6.616, *p* = 0.0001] in males and females. Females drank more water than males [two-way ANOVA: F_(1,21)_ = 17.67, *p* = 0.0004]. There was no significant interaction effect between sex and time [two-way ANOVA: *F*_(14,2.93)_ = 1.608, *p* = 0.0761]. **(F)** Total water consumption for males and females. **(G)** Plot showing the alcohol preferences as percentage of total alcohol consumption relative to total fluid consumption for each concentration of alcohol. Alcohol % had an effect on preference [two-way ANOVA: *F*_(3, 66)_ = 10.01, *p* < 0.0001]. There was no sex effect [two-way ANOVA: *F*_(1, 22)_ = 2.006, *p* = 0.1707].

Mice underwent two-bottle choice voluntary drinking and were then divided into two groups: an acute abstinence and a protracted abstinence group. The acute abstinence group went through 24 h of alcohol abstinence following the last drinking session while the protracted abstinence group went through 3 weeks of abstinence from alcohol. Both groups are shown together in Figure 1 to display drinking patterns, and then analyzed separately for all other experiments. To ensure only true drinking values were analyzed, sessions with malfunctions like bottle leaks were excluded from analysis. As alcohol concentration increased over time, so did the amount of alcohol consumed in individual mice who were exposed to alcohol (Figure 1A). Total water consumption remained mostly steady throughout the 15 drinking sessions, however, a time effect was seen at sessions 11, 13, 14 and 15 (Figure 1B). When analyzed by sex, females exhibited a greater amount of alcohol consumption than males over time (Figures 1C, D). There was an effect of time on both males and females and an interaction of sex and time was also found. Water consumption also differed in females and males, with females showing an overall higher intake (Figures 1E, F). For the first 6 sessions, the alcohol concentration was increased from 3% to 6%–10%, with mice getting access to each concentration for 2 drinking sessions. The concentration was then increased to 20% for the remaining sessions. Overall, there was no difference in the preference for alcohol over water between males and females (Figure 1G). However, the concentration of alcohol had an effect on preference.

### Acute abstinence from alcohol produced more proteome changes than protracted abstinence

To determine distinct modifications in mouse brain regions due to alcohol abstinence, we dissected the striatum into three brain regions: DMS, DLS and NAc. We then performed mass spectrometry and analyzed proteins that were differentially expressed as a product of alcohol drinking with different lengths of post-drinking abstinence. Protein analyses were done within each brain region, sex, and abstinence group.

**FIGURE 2:**
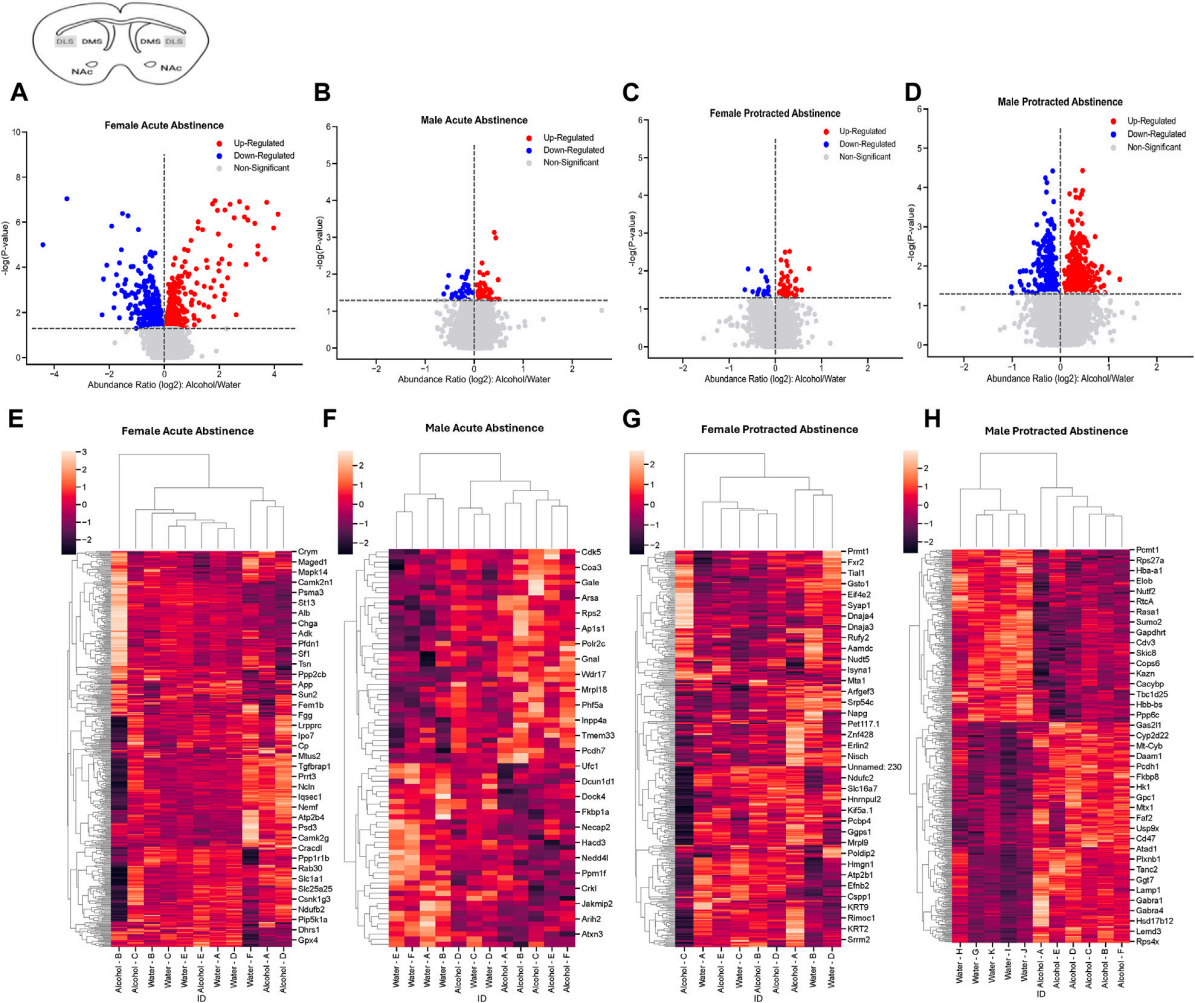
Differentially abundant proteins in the dorsolateral striatum of mice that were exposed to alcohol and underwent acute and protracted abstinence. Volcano plots display differential protein abundance in acute abstinence for **(A)** females (*n* = 11) and **(B)** males (*n* = 12). Individual increased and decreased proteins that reached the level of significance are shown in red and blue, respectively. **(C,D)** show clusterplots and dendrograms of proteins with a significant change in abundance due to acute alcohol abstinence. Some representative GeneIDs are provided on the right. Volcano plots display differential protein abundance in protracted abstinence for **(E)** females (*n* = 9) and **(F)** males (*n* = 11). Individual increased and decreased proteins that reached the level of significance are shown in red and blue, respectively. **(G,H)** show clusterplots and dendrograms of proteins with a significant change in abundance due to protracted alcohol abstinence. Some representative GeneIDs are provided on the right.

In the DLS of female mice that went through acute abstinence, a total of 570 proteins’ abundances were significantly altered, with 311 increased and 259 decreased proteins (Figure 2A). In males, fewer proteins were significantly differentially abundant; 42 proteins were increased and 36 proteins were decreased (Figure 2B). Clusterplots with dendrograms show significantly abundant proteins in the DLS of acute abstinence males (Figure 2C) and females (Figure 2D) based on their similarity. Proteins in alcohol and water drinkers were seen to be most dissimilar in females, however there was no clear distinction of proteins in males.

The opposite trend was seen in the protein abundances for males and females that underwent protracted abstinence, as males showed a larger number of proteins with significantly altered abundance. Females had 20 increased and 61 decreased proteins (Figure 2E) while males had 268 increased and 342 decreased proteins (Figure 2F). The clusterplots also displayed an opposing trend to the acute group, where protein abundances for male alcohol and water drinkers were most dissimilar but there was not a consistent pattern for females (Figures 2G, H). The top 10 increased and decreased proteins in the DLS in each sex and abstinence group are listed in Table 1.

**TABLE 1:** Top 10 increased and decreased proteins in the dorsolateral striatum within each sex and alcohol abstinence group.

Accession #	Description	*p*-value	Accession #	Description	*p*-value
Top 10 Upregulated in acute female			Top 10 Upregulated in acute male		
A0JP43	EF-hand calcium-binding domain-containing protein 5	4.43E-07	A0A1D5RML8	DNA-directed RNA polymerase II subunit RPB3	0.0469
Q9EST4	Proteasome assembly chaperone 2	1.82E-06	Q66L47	Guanine nucleotide binding protein, *alpha* stimulating, olfactory type	0.0142
Q9CWT6	Probable ATP-dependent RNA helicase DDX28	1.31E-07	P28867	Protein kinase C delta type	0.0010
E9PUQ3	NKAP domain-containing 1	4.49E-05	Q9WTW5	Solute carrier family 22 member 3	0.0465
A0A0U1RP06	DNA repair protein-complementing XP-C cells homolog (Fragment)	1.12E-05	H3BLI8	WD repeat domain 17	0.0007
V9GXG1	Protein SLFN14	2.54E-05	Q80YS4	receptor protein-tyrosine kinase	0.0481
E9Q099	Oviduct-specific glycoprotein	1.12E-06	Q5DU31	Interactor protein for cytohesin exchange factors 1	0.0306
Q8BTK5	SET and MYND domain-containing protein 4	8.09E-07	Q9WV27	Sodium/potassium-transporting ATPase subunit *alpha*-4	0.0264
Q5FW96	Nuclear receptor subfamily 1 group I member 3	8.06E-07	C3S7Q5	Ojoplano variant A	0.0325
Q9ER47	Potassium voltage-gated channel subfamily H member 7	2.28E-07	E9QNX9	Tyrosine-protein kinase receptor	0.0437
Top 10 downregulated in acute female			Top 10 downregulated in acute male		
Q68FM4	protein-tyrosine-phosphatase	1.00E-05	Q91VJ5	Polyglutamine-binding protein 1	0.0341
V9GWU7	non-specific serine/threonine protein kinase	9.07E-08	Q8BN72	ATP-dependent RNA helicase DHX29	0.0223
F8WHT3	Protein PRRC2B	1.25E-02	O54983	Ketimine reductase mu-crystallin	0.0108
Q8BM75	AT-rich interactive domain-containing protein 5B	3.29E-04	F8VQ87	5′-3′ exoribonuclease 1	0.0440
B1AWL5	Zinc finger protein 462	8.11E-05	Q80UF4	Serologically defined colon cancer antigen 8 homolog	0.0353
P97427	Dihydropyrimidinase-related protein 1	1.50E-06	Q9Z0H3	SWI/SNF-related matrix-associated actin-dependent regulator of chromatin subfamily B member 1	0.0371
P03893	NADH-ubiquinone oxidoreductase chain 2	6.07E-03	B1ART2	Vacuolar protein sorting 13D	0.0322
Q9R0H2	Endomucin	1.44E-03	Q6P3B2	UBA-like domain-containing protein 1	0.0420
Q91XX1	Protocadherin gamma C3	2.12E-04	Q9R0L6	Pericentriolar material 1 protein	0.0284
F2Z3Y4	GATOR complex protein NPRL3	6.24E-04	Q8BZN6	Dedicator of cytokinesis protein 10	0.0452
Top 10 upregulated in protracted female			Top 10 upregulated in protracted male		
E9QJY0	Cationic amino acid transporter 2	0.0087	Q9R0M4	Podocalyxin	2.92E-05
A0A286YCQ5	Nidogen-2 (Fragment)	0.0314	E9Q968	Pituitary adenylate cyclase-activating polypeptide type I receptor	2.92E-05
D6RCU5	ATP-binding cassette sub-family C member 12	0.0316	P47759	Neuronal vesicle trafficking-associated protein 2	2.92E-05
Q60675	Laminin subunit *alpha*-2	0.0164	Q8JZL2	Melanin-concentrating hormone receptor 1	2.92E-05
E9Q4D0	Proprotein convertase subtilisin/kexin type 6	0.0429	Q9DCF9	Translocon-associated protein subunit gamma	2.92E-05
Q9D6Y4	BLOC-1-related complex subunit 8	0.0177	B2RXS4	Plexin-B2 OS = *Mus musculus*	2.92E-05
A0A494B933	Protein phosphatase 1 regulatory subunit 14 (Fragment)	0.0288	E9PW43	Predicted pseudogene 10320	3.50E-05
Q7TNL9	Coiled-coil-helix-coiled-coil-helix domain-containing 10	0.0463	Q8VEA4	Mitochondrial intermembrane space import and assembly protein 40	3.50E-05
Q9D0J8	Parathymosin	0.0380	Q80V26	Golgi-resident adenosine 3′,5′-bisphosphate 3′-phosphatase	4.33E-05
Q9QZD8	Mitochondrial dicarboxylate carrier	0.0421	Q5DTL9	Sodium-driven chloride bicarbonate exchanger	4.33E-05
Top 10 downregulated in protracted female			Top 10 downregulated in protracted male		
F8VPZ3	Ubiquitinyl hydrolase 1	0.0307	A0A1L1STC5	Guanine nucleotide-binding protein subunit beta-5	0.0332
A0A286YDC5	Mitochondrial intermediate peptidase	0.0089	A0A8Q0Q6H9	Steroid receptor RNA activator 1	0.0476
Q8BGT5	Alanine aminotransferase 2	0.0348	O35945	Aldehyde dehydrogenase, cytosolic 1	0.0139
V9GXP8	ELKS/Rab6-interacting/CAST family member 1	0.0443	P63300	Selenoprotein W OS = *Mus musculus*	0.0248
E9Q7M2	TSC22 domain family, member 2	0.0368	E9Q448	Tropomyosin *alpha*-1 chain	0.0296
Q80WM4	Hyaluronan and proteoglycan link protein 4	0.0342	G3X8Y1	Tripartite motif-containing 55	0.0133
Q920P3	BMP/retinoic acid-inducible neural-specific protein 1	0.0302	Q9Z0J0	NPC intracellular cholesterol transporter 2	0.0391
Q6P5U7	NACHT and WD repeat domain-containing protein 2	0.0100	Q7M6Z4	Kinesin-like protein KIF27	0.0132
A0A0J9YUM2	Ras/Rap GTPase-activating protein SynGAP	0.0393	G3UWX9	Small ubiquitin-related modifier 3	0.0493
B7ZC46	Septin	0.0486	P08228	Superoxide dismutase [Cu-Zn]	0.0448

**FIGURE 3:**
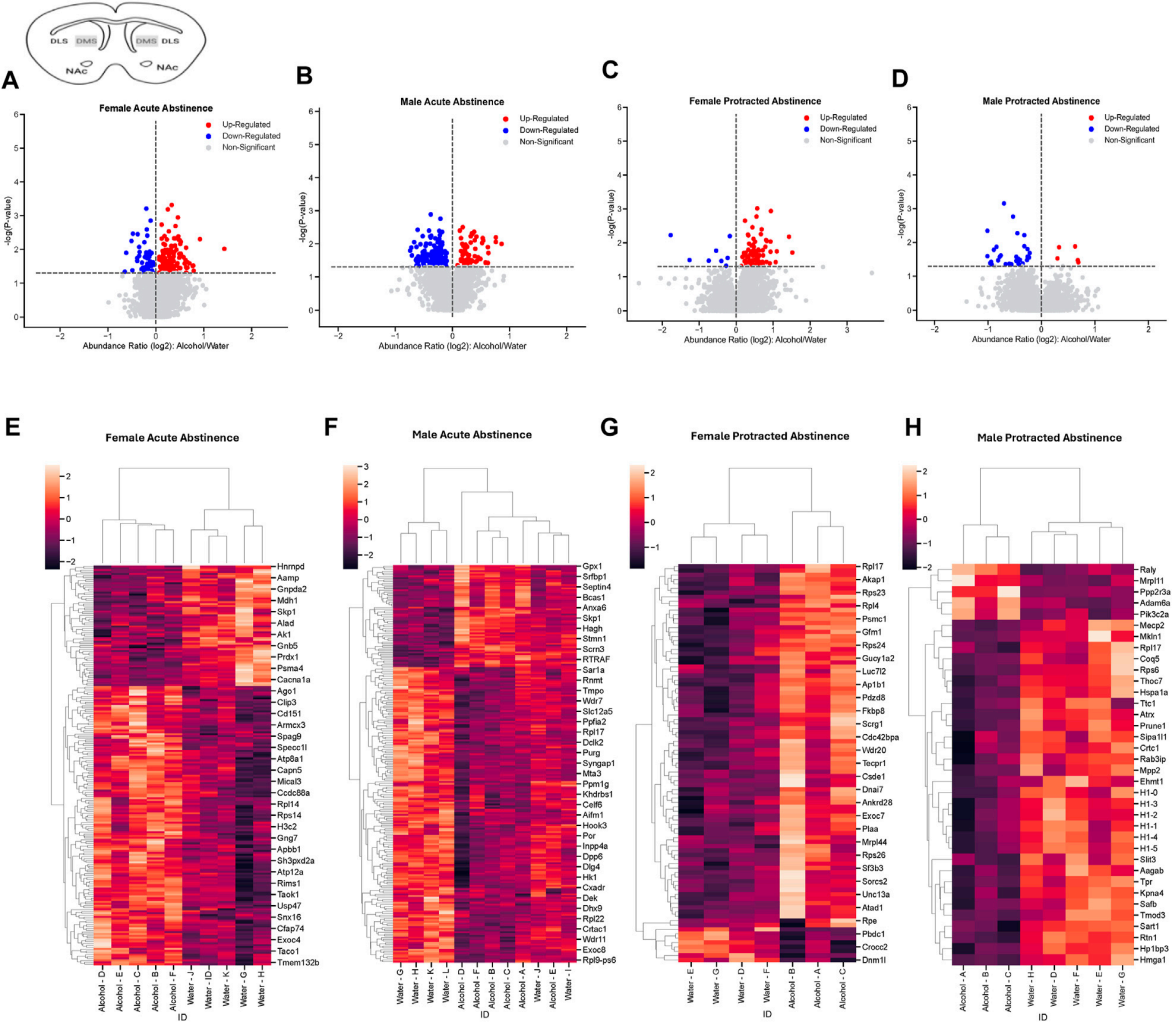
Differentially abundant proteins in the dorsomedial striatum of mice that were exposed to alcohol and underwent acute and protracted abstinence. Volcano plots display differential protein abundance in acute abstinence for **(A)** females (*n* = 10) and **(B)** males (*n* = 12). Individual increased and decreased proteins that reached the level of significance are shown in red and blue, respectively. **(C,D)** show clusterplots and dendrograms of proteins with a significant change in abundance due to acute alcohol abstinence. Some representative GeneIDs are provided on the right. Volcano plots display differential protein abundance in protracted abstinence for **(E)** females (*n* = 7) and **(F)** males (*n* = 8). Individual increased and decreased proteins that reached the level of significance are shown in red and blue, respectively. **(G,H)** show clusterplots and dendrograms of proteins with a significant change in abundance due to protracted alcohol abstinence. Some representative GeneIDs are provided on the right.

Next, we assessed the proteome changes in the DMS. The total number of proteins that had significantly altered abundance as a result of alcohol drinking and abstinence were similar between males and females in the acute abstinence group. However, the pattern of increased abundance relative to decreased abundance were opposite between each sex. In female mice, there were 148 proteins increased and 64 proteins decreased (Figure 3A) while males had 59 increased and 171 decreased proteins (Figure 3B). In analyzing clustering of differentially abundant proteins, we saw that alcohol and water drinkers were distinctly dissimilar in both females and males (Figures 3C, D). For mice that experienced protracted abstinence, total protein abundance was lower than those that went through acute abstinence. There were 83 increased and 8 decreased proteins in females (Figure 3E), and 5 increased and 31 decreased in males (Figure 3F). Analyses of the clustering data show that protein abundances were most dissimilar between the water and alcohol groups in all animals (Figures 3G, H). Table 2 shows the top 10 increased and decreased proteins in the DMS within each sex and abstinence group.

**TABLE 2:** Top 10 increased and decreased proteins in the dorsomedial striatum within each sex and alcohol abstinence group.

Accession #	Description	*p*-value	Accession #	Description	*p*-value
Top 10 upregulated in acute female			Top 10 upregulated in acute male		
P84228	Histone H3.2	0.0101	Q6P1B9	Bin1 protein	0.006708
Q61102	Iron-sulfur clusters transporter ABCB7, mitochondrial	0.0064	Q8JZP2	Synapsin-3	0.007959
Q6ZQ08	CCR4-NOT transcription complex subunit 1	0.0088	P08551	Neurofilament light polypeptide	0.036803
A0A1Y7VMH3	Serine/threonine-protein kinase VRK1	0.0190	P02802	Metallothionein-1	0.037229
B1AV77	Aldehyde dehydrogenase	0.0126	Q8R3P0	Aspartoacylase	0.012594
P61255	60S ribosomal protein L26	0.0378	A0A0R4J036	Neurofilament medium polypeptide	0.037806
P12970	60S ribosomal protein L7a	0.0368	Q9ESM3	Hyaluronan and proteoglycan link protein 2	0.018964
Q9CR57	60S ribosomal protein L14	0.0372	Q8BR63	Protein FAM177A1	0.006408
Q9R0M0	Cadherin EGF LAG seven-pass G-type receptor 2	0.0080	P19246	Neurofilament heavy polypeptide	0.008773
B1AR17	DNA helicase	0.0067	Q9JK88	Serpin I2	0.010082
Top 10 downregulated in acute female			Top 10 downregulated in acute male		
B9EHT4	CAP-Gly domain-containing linker protein 3	0.0090	Q61136	Serine/threonine-protein kinase PRP4 homolog	0.0161
Q3U0M1	Trafficking protein particle complex subunit 9	0.0356	D3Z2V6	Ras-related protein M-Ras (Fragment)	0.0130
P10518	Delta-aminolevulinic acid dehydratase	0.0347	P63213	Guanine nucleotide-binding protein G(I)/G(S)/G(O) subunit gamma-2	0.0224
D3YWK6	Thiopurine S-methyltransferase (Fragment)	0.0242	A2AI21	Glutamate receptor	0.0089
Q9DBB8	Trans-1,2-dihydrobenzene-1,2-diol dehydrogenase	0.0259	Q8R555	Cartilage acidic protein 1	0.0311
Q9D711	Pirin	0.0312	P23818	Glutamate receptor 1	0.0124
Q3UHX2	28 kDa heat- and acid-stable phosphoprotein	0.0302	A2ALL9	Calcium-transporting ATPase	0.0283
B2RUG9	Adenomatosis polyposis coli	0.0432	P0DN34	NADH dehydrogenase [ubiquinone] 1 beta subcomplex subunit 1	0.0327
Q62048	Astrocytic phosphoprotein PEA-15	0.0050	Q8BG51	Mitochondrial Rho GTPase 1	0.0450
Q8K449	ATP-binding cassette sub-family A member 9	0.0096	Q9CPR4	60S ribosomal protein L17	0.0038
Top 10 upregulated in protracted female			Top 10 upregulated in protracted male		
Q9JK88	Serpin I2	0.0101	Q61194	Phosphatidylinositol 4-phosphate 3-kinase C2 domain-containing subunit *alpha*	0.0388
Q8BR63	Protein FAM177A1	0.0064	B2RSY5	A disintegrin and metallopeptidase domain 6	0.0337
P19246	Neurofilament heavy polypeptide	0.0088	Q9CQF0	39S ribosomal protein L11, mitochondrial	0.0132
Q9ESM3	Hyaluronan and proteoglycan link protein 2	0.0190	Q64012	RNA-binding protein Raly	0.0139
Q8R3P0	Aspartoacylase	0.0126	B2RXC8	3222402P14Rik protein	0.0298
A0A0R4J036	Neurofilament medium polypeptide	0.0378	Q91Z38	Tetratricopeptide repeat protein 1	0.0206
P08551	Neurofilament light polypeptide	0.0368	D3YXK2	Scaffold attachment factor B1	0.0278
P02802	Metallothionein-1	0.0372	Q8BIW1	Exopolyphosphatase PRUNE1	0.0158
Q8JZP2	Synapsin-3	0.0080	Q9WV34	MAGUK p55 subfamily member 2	0.0265
Q6P1B9	Bin1 protein	0.0067	Q7M739	Nucleoprotein TPR	0.0334
Top 10 downregulated in protracted female			Top 10 downregulated in protracted male		
Q61136	Serine/threonine-protein kinase PRP4 homolog	0.0161	P43274	Histone H1.4	0.0256
D3Z2V6	Ras-related protein M-Ras (Fragment)	0.0130	Q61687	Transcriptional regulator ATRX	0.0045
P63213	Guanine nucleotide-binding protein subunit gamma-2	0.0224	O89050	Muskelin	0.0410
A2AI21	Glutamate receptor	0.0089	P10922	Histone H1.0	0.0378
Q8R555	Cartilage acidic protein 1	0.0311	A0A0A6YWA0	Histone-lysine N-methyltransferase EHMT1 (Fragment)	0.0444
P23818	Glutamate receptor 1	0.0124	Q9JHJ0	Tropomodulin-3	0.0165
A2ALL9	Calcium-transporting ATPase	0.0283	P43276	Histone H1.5	0.0135
P0DN34	NADH dehydrogenase [ubiquinone] 1 beta subcomplex subunit 1	0.0327	P43277	Histone H1.3	0.0340
Q8BG51	Mitochondrial Rho GTPase 1	0.0450	Q9CXI0	2-methoxy-6-polyprenyl-1,4-benzoquinol methylase, mitochondrial	0.0267
Q9CPR4	60S ribosomal protein L17	0.0038	P43275	Histone H1.1	0.0245

**FIGURE 4:**
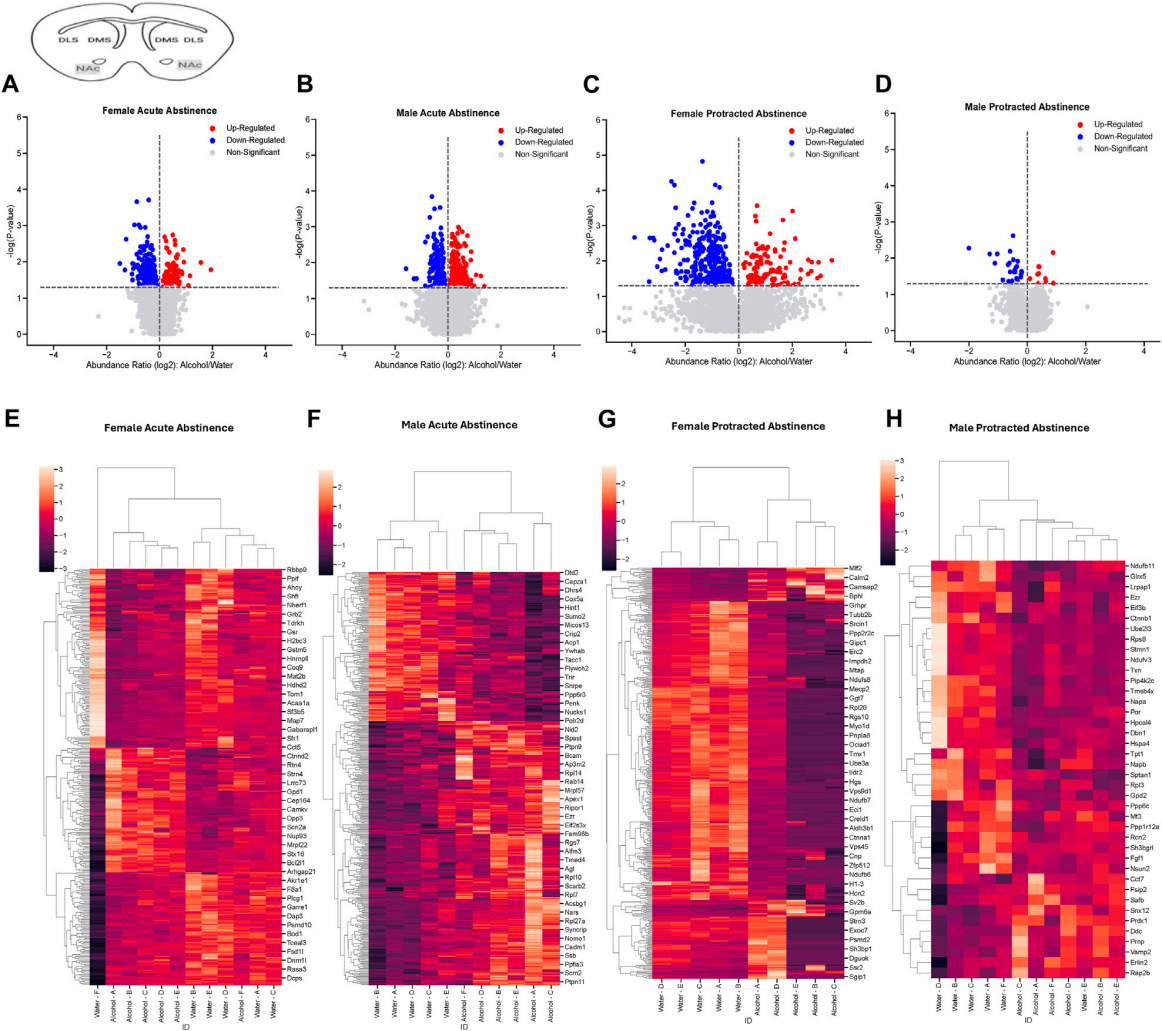
Differentially abundant proteins in the nucleus accumbens of mice that were exposed to alcohol and underwent acute and protracted abstinence. Volcano plots display differential protein abundance in acute abstinence for **(A)** females (*n* = 12) and **(B)** males (*n* = 11). Individual increased and decreased proteins that reached the level of significance are shown in red and blue, respectively. **(C,D)** show clusterplots and dendrograms of proteins with a significant change in abundance due to acute alcohol abstinence. Some representative GeneIDs are provided on the right. Volcano plots display differential protein abundance in protracted abstinence for **(E)** females (*n* = 10) and **(F)** males (*n* = 12). Individual increased and decreased proteins that reached the level of significance are shown in red and blue, respectively. **(G,H)** show clusterplots and dendrograms of proteins with a significant change in abundance due to protracted alcohol abstinence. Some representative GeneIDs are provided on the right.

The final brain region we assessed was the NAc. In mice that were exposed to acute abstinence, 230 increased and 98 decreased proteins were identified in females (Figure 4A). In males with acute abstinence, there were slightly more proteins affected with 273 increased and 154 decreased (Figure 4B). Figures 4C, D show a similar pattern as in the DMS with water and alcohol drinking largely driving the clustering. Female mice that were exposed to protracted abstinence had 322 increased and 121 decreased proteins (Figure 4E). However, the total amount of significantly abundant proteins in males was much lower, with 10 being increased and 39 decreased (Figure 4F). Figures 4G, H show that alcohol and water drinking were most dissimilar in males and females. The top increased and decreased proteins within each sex and abstinence group can be found in Table 3.

**TABLE 3:** Top 10 increased and decreased proteins in the nucleus accumbens within each sex and alcohol abstinence group.

Accession #	Description	*p*-value	Accession #	Description	*p*-value
Top 10 Upregulated in Acute Female			Top 10 upregulated in acute male		
Q3TXX4	Vesicular glutamate transporter 1	0.0415	Q91Z49	UAP56-interacting factor	0.0461
Q3UHL1	CaM kinase-like vesicle-associated protein	0.0362	Q80TN5	Palmitoyltransferase ZDHHC17	0.0086
Q5DQR4	Syntaxin-binding protein 5-like	0.0188	Q8R0W0	Epiplakin	0.0315
Q9CQW2	ADP-ribosylation factor-like protein 8B	0.0219	Q8CAL5	Glypican-5	0.0050
P62245	40S ribosomal protein S15a	0.0046	Q64378	Peptidyl-prolyl cis-trans isomerase FKBP5	0.0475
B2RWC4	Gm88 protein	0.0103	Q8BHC4	Dephospho-CoA kinase domain-containing protein	0.0430
Q9QXG2	Rab proteins geranylgeranyltransferase component A 1	0.0449	B7ZCF4	Lysosomal acid phosphatase (Fragment)	0.0448
D3YVU3	Centrosomal protein of 164 kDa	0.0245	Q62313	Trans-Golgi network integral membrane protein 1	0.0223
D3Z5I9	RNA-binding protein 33	0.0105	Q8BYW9	EGF domain-specific O-linked N-acetylglucosamine transferase	0.0239
Q6GQS1	Calcium-binding mitochondrial carrier protein SCaMC-3	0.0166	Q9QWW1	Homer protein homolog 2	0.0454
Top 10 downregulated in acute female			Top 10 downregulated in acute male		
A0A498WGR4	Galanin peptides	0.0112	E9PZ08	FYN-binding protein 2	0.0149
P97825	Jupiter microtubule associated homolog 1	0.0168	F6VCP8	FLYWCH family member 2 (Fragment)	0.0284
G3UW82	Myosin, heavy polypeptide 2, skeletal muscle, adult	0.0024	Q9WUE4	GATOR complex protein NPRL2	0.0281
Q8BK72	28S ribosomal protein S27, mitochondrial	0.0218	A0A0N4SVP9	SWI/SNF-related matrix-associated actin-dependent regulator of chromatin subfamily A-containing DEAD/H box 1 (Fragment)	0.0027
F6RPX5	*Alpha*-tubulin N-acetyltransferase 1 (Fragment)	0.0316	Q6P1B9	Bin1 protein	0.0445
P20934	Protein EVI2A	0.0112	P09240	Cholecystokinin	0.0019
P09240	Cholecystokinin	0.0154	Q8BG58	Transmembrane prolyl 4-hydroxylase	0.0471
Q06138	Calcium-binding protein 39	0.0115	Q6W8Q3	Purkinje cell protein 4-like protein 1	0.0261
Q9DD18	D-aminoacyl-tRNA deacylase 1	0.0010	Q9D735	Telomerase RNA component interacting RNase	0.0189
A2A4A6	1-phosphatidylinositol 4,5-bisphosphate phosphodiesterase gamma	0.0062	Q60698	Ski oncogene	0.0278
Top 10 Upregulated in protracted female			Top 10 upregulated in protracted male		
P39429	TNF receptor-associated factor 2	0.0328	P80313	T-complex protein 1 subunit eta	0.0372
E9Q3B2	Pogo transposable element with KRAB domain	0.0180	Q8BFZ9	Erlin-2	0.0283
Q9DC07	LIM zinc-binding domain-containing Nebulette	0.0237	P35700	Peroxiredoxin-1	0.0497
F7CPX0	SH3-containing GRB2-like protein 3-interacting protein 1 (Fragment)	0.0095	A2ARZ3	Fibrous sheath-interacting protein 2	0.0172
F6T8K1	Izumo sperm-egg fusion protein 3 (Fragment)	0.0196	Q6ZWQ5	Sorting nexin-12	0.0267
Q99PV8	B-cell lymphoma/leukemia 11B	0.0109	P04925	Major prion protein	0.0175
P35487	Pyruvate dehydrogenase E1 component subunit *alpha*, testis-specific form, mitochondrial	0.0311	P61226	Ras-related protein Rap-2b	0.0370
A0A571BET0	Voltage-dependent P/Q-type calcium channel subunit *alpha*	0.0111	D3YXK2	Scaffold attachment factor B1	0.0433
Q8BJR7	Centrosomal protein of 97 kDa	0.0268	Q5SUV9	Aromatic-L-amino-acid decarboxylase (Fragment)	0.0071
E9Q350	BAI1-associated protein 3	0.0097	B0QZN5	Vesicle-associated membrane protein 2	0.0491
Top 10 downregulated in protracted female			Top 10 downregulated in protracted male		
Q8BTF8	RNA-binding Raly-like protein	0.0022	Q80Y14	Glutaredoxin-related protein 5, mitochondrial	0.0053
E9Q1K3	*Alpha*-adducin	0.0386	A0A338P7E5	Ubiquitin-conjugating enzyme E2 L3	0.0077
P83887	Tubulin gamma-1 chain	0.0023	Q3U422	NADH dehydrogenase [ubiquinone] flavoprotein 3, mitochondrial	0.0139
Q9JHR7	Insulin-degrading enzyme	0.0023	P20065	Thymosin beta-4	0.0077
O08582	GTP-binding protein 1	0.0026	P28184	Metallothionein-3	0.0401
P56388	Cocaine- and amphetamine-regulated transcript protein	0.0147	P54227	Stathmin	0.0152
Q80VQ0	Aldehyde dehydrogenase family 3 member B1	0.0089	Q9DBR7	Protein phosphatase 1 regulatory subunit 12A	0.0439
D6REG4	Glyoxylate reductase/hydroxypyruvate reductase	0.0194	P61148	Fibroblast growth factor 1	0.0138
Q9CRD4	Dysbindin domain-containing protein 2	0.0048	Q91XU3	Phosphatidylinositol 5-phosphate 4-kinase type-2 gamma	0.0065
A0A1Y7VN19	Dehydrogenase/reductase SDR family member 7 (Fragment)	0.0178	P62242	40S ribosomal protein S8	0.0242

### Overlap analyses shows distinct protein changes between striatal subregions and abstinence lengths

**FIGURE 5:**
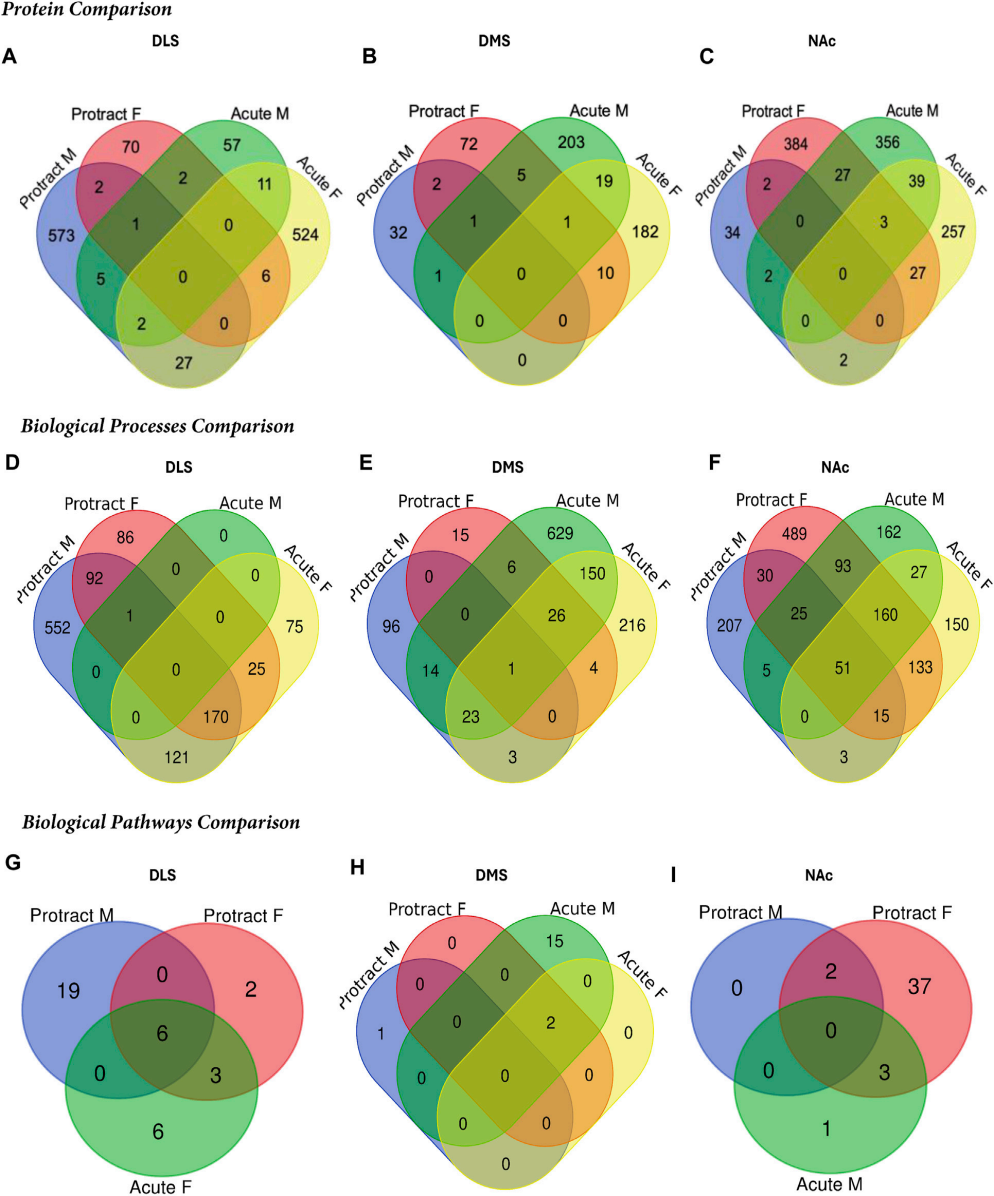
Overlap in the proteome of animals with voluntary alcohol consumption. Venn diagrams show the protein comparison between the protracted and acute abstinence and sex groups in the **(A)** DLS, **(B)** DMS, and **(C)** NAc. Venn diagrams also show a comparison of gene ontology biological processes in the **(D)** DLS, **(E)** DMS and **(F)** NAc and KEGG biological pathways in the **(G)** DLS, **(H)** DMS and **(I)** NAc. Any missing categories indicate a lack of identified significant biological processes and pathways. Protract M: protracted abstinence male; Protract **(F)** protracted abstinence female; Acute M: acute abstinence male; Acute F: acute abstinence female.

To determine if there were overlapping proteins between each sex and abstinence period within each brain region, we compared all significantly abundant proteins within each of the DLS, DMS and NAc regions (Figures 5A–C; Table 4). There was no protein within any brain region that was commonly affected between each sex and abstinence period. In the DLS, there were 11 overlapping proteins between acute abstinence males and females, and 2 between protracted abstinence males and females. In the DMS, 19 overlapping proteins were identified between acute abstinence males and females and 2 between protracted mice. Finally, in the NAc, there were 39 overlapping proteins between acute abstinence groups and 2 overlapping proteins between protracted groups.

**TABLE 4:** Overlapping protein abundance in striatal subregions.

DLS		DMS				NAc				
Protracted female	Acute	Protracted female	Acute			Protracted female	Acute			
Vs. Male	Female Vs. Male	Vs. Male	Female Vs. Male			Vs. Male	Female Vs. Male			
Slc25a10	Coa3	Rps6	Atp8a1	Ptn		Napb	Dnm1	Dhrs4	Thoc7	Rab2a
Hdhd2	Sars2	Sart1	Rpl38	Cfl1		Fgf1	Pgm2l1	Cask	Dnm1l	Cck
	Fth1		Negr1	Exoc8			Camk1d	Pyurf	Mprip	Cadm1
	Trip4		Rab5b	Dclk2			Ddx46	Thtpa	Bckdha	Rasa3
	Gale		Kcnd2	Sbf1			Cox6a1	Strn4	Tfg	Dcps
	Fkbp5		Lrp1	Rps19			Ak1	Cox6b1	Nme2	Czib
	Pqbp1		Gk	Aifm1			Rps28	Shfl	Crtc1	
	Cdk5		Rps14	Skp1			Nars1	Rabif	Grpel1	
	Arih2		Rps11				Dctn6	Bad	Ctnnd2	
	Camk1		Nomo1				Acp1	Rras2	Rgs7	
	Necap		Ak1				Rpl15	Atp6v1f	Ube2m	

### Biological process and pathway analyses reveal sex and abstinence length effects of alcohol exposure in the DLS, DMS and NAc

To further investigate proteins that were affected by alcohol drinking followed by abstinence, we conducted a gene ontology analysis for biological processes. The first brain region we looked at was the DLS. For mice exposed to acute abstinence, 464 biological processes were affected in females and 3 in males. For those exposed to protracted abstinence, 469 biological processes were modified in females and 1,051 in males. The top biological processes (*p* < 0.05) in the DLS are displayed in Table 5.

**TABLE 5:** Top 10 biological processes in the DLS.

Description	Adjusted *p*-value	Description	Adjusted *p*-value
Protracted male		Protracted female	
Regulation of transport	2.31E-29	Central nervous system development	3.43E-11
Vesicle-mediated transport	3.04E-27	Small molecule metabolic process	9.91E-10
Establishment of protein localization	1.32E-26	Organonitrogen compound biosynthetic process	1.02E-09
Intracellular transport	2.85E-26	Positive regulation of protein localization	1.21E-08
Positive regulation of cellular component organization	3.46E-23	Protein transport	1.88E-08
Membrane organization	1.24E-21	Purine-containing compound metabolic process	3.23E-08
Protein transport	3.48E-21	Neuron projection development	3.61E-08
Plasma membrane bounded cell projection organization	5.08E-21	Localization within membrane	3.61E-08
Cell projection organization	7.09E-21	Plasma membrane bounded cell projection organization	3.64E-08
Cell junction organization	3.97E-20	Energy derivation by oxidation of organic compounds	4.00E-08
Acute male		Acute female	
Positive regulation of protein metabolic process	0.00154	Vesicle-mediated transport	1.38E-16
		Establishment of protein localization	2.85E-15
		Protein transport	4.11E-15
		Organonitrogen compound biosynthetic process	6.98E-13
		Cell junction organization	2.57E-12
		Localization within membrane	4.16E-11
		Neuron projection development	6.79E-11
		Amide biosynthetic process	1.42E-10
		Synapse organization	1.42E-10
		Cellular component morphogenesis	3.10E-10

Only biological processes with an adjusted *p*-value < 0.05 are shown.

In the DMS, 980 biological processes were affected in acute females, 1,344 in acute males, 140 in protracted females, and 452 in protracted males. In the NAc, 871 processes were significantly modified in acute females, 873 in acute males, 1,435 in protracted females, and 918 in protracted males. Tables 6, 7 list the top 10 processes in the DMS and NAc respectively. We used the online tool Revigo to simplify the lengthy list of biological processes. The simplified lists for each striatal subregion and abstinence length can be found in the Supplementary Material. We also assessed overlapping biological processes between striatal subregions and lengths of abstinence. In the DLS, 1 process was found to overlap between acute males, protracted males and protracted females and 170 between acute females, protracted females and protracted males (Figure 5D). In the DMS, 1 biological process was found to overlap all groups (Figure 5E). Finally, 51 processes were found to overlap all groups in the NAc (Figure 5F). Overlapping biological processes can be found in Supplementary Table S2.

**TABLE 6:** Top 10 biological processes in the DMS.

Description	Adjusted *p*-value	Description	Adjusted *p*-value
Protracted male		Protracted female	
Nucleosome assembly	1.61E-10	Cytoplasmic translation	5.69E-12
Nucleosome organization	4.57E-10	Peptide metabolic process	7.85E-10
Chromosome condensation	5.76E-09	Amide metabolic process	1.26E-08
Negative regulation of DNA recombination	1.00E-08	Translation at postsynapse	7.56E-08
Protein-DNA complex assembly	2.51E-08	Translation at synapse	7.56E-08
Chromatin remodeling	3.90E-08	Translation at presynapse	7.56E-08
Chromatin organization	2.04E-07	Translation	8.13E-08
Heterochromatin organization	4.95E-07	Peptide biosynthetic process	1.15E-07
Protein-DNA complex organization	5.03E-07	Amide biosynthetic process	1.24E-07
Regulation of DNA recombination	1.99E-06	Ribosome biogenesis	1.54E-05
Acute male		Acute female	
Cytoplasmic translation	3.22E-18	Cytoplasmic translation	1.98E-14
Cellular localization	3.22E-18	Translation at presynapse	3.66E-14
Cellular component organization or biogenesis	3.22E-18	Translation at synapse	3.66E-14
Localization	2.37E-17	Translation at postsynapse	3.66E-14
Cellular component organization	1.54E-16	Amide biosynthetic process	2.32E-10
Organelle organization	9.36E-16	Translation	4.02E-10
Establishment of localization	2.38E-14	Peptide biosynthetic process	7.32E-10
Transport	2.63E-13	Amide metabolic process	2.20E-09
Regulation of biological quality	9.91E-13	Peptide metabolic process	3.91E-09
Regulation of localization	1.58E-12	Organonitrogen compound biosynthetic process	1.28E-08

Only biological processes with an adjusted *p*-value < 0 .05 are shown.

**TABLE 7:** Top 10 biological processes in the NAc.

Description	Adjusted *p*-value	Description	Adjusted *p*-value
Protracted Male		Protracted female	
Regulation of cellular component biogenesis	0.00032	Cellular component organization or biogenesis	1.41E-24
Regulation of protein-containing complex assembly	0.00100	Localization	1.92E-24
Negative regulation of supramolecular fiber organization	0.00404	Organelle organization	2.92E-24
Negative regulation of cytoskeleton organization	0.00404	Cellular localization	2.52E-23
Regulation of synaptic vesicle priming	0.00620	Cellular component organization	2.67E-23
Regulation of protein polymerization	0.00620	Establishment of localization	6.26E-23
Synaptic vesicle cycle	0.00659	Transport	6.83E-23
Actin filament organization	0.00678	Cell junction organization	1.42E-20
Negative regulation of protein polymerization	0.00808	Establishment of localization in cell	4.45E-20
Protein-containing complex disassembly	0.00808	Regulation of cellular component organization	1.91E-19
Acute male		Acute female	
Cytoplasmic translation	1.33E-23	Organelle organization	9.10E-16
Organonitrogen compound metabolic process	5.06E-22	Cellular localization	3.52E-15
Translation	5.50E-19	Localization	1.21E-13
Peptide biosynthetic process	1.31E-18	Organonitrogen compound metabolic process	1.03E-12
Protein metabolic process	1.31E-18	Establishment of localization in cell	1.34E-12
Peptide metabolic process	1.31E-18	Establishment of localization	9.67E-12
Cellular localization	3.23E-17	Transport	7.93E-11
Establishment of localization in cell	9.56E-17	Cellular component organization or biogenesis	8.34E-11
Amide biosynthetic process	1.40E-16	Protein localization	2.03E-10
Cellular process	4.68E-16	Cellular component organization	2.03E-10

Only biological processes with an adjusted *p*-value < 0.05 are shown.

We next investigated further to assess the biological pathways that significant overlapping proteins were involved in. We conducted this search using the KEGG biological pathways database. This revealed that there were pathways enriched by alcohol consumption followed by acute and protracted abstinence. In the DLS, 15 pathways were affected in acute females, 10 in acute males, 11 in protracted females and 25 in protracted males. The top 10 biological pathways in the DLS are displayed in Table 8. Overlapping biological pathways were also identified. In the DLS, 6 pathways overlapped between the protracted males and females and acute females and 3 between acute and protracted females (Figure 5G; Supplementary Table S3). We conducted the same search in the DMS and saw that less biological pathways were affected in all groups except acute males, when compared to the DLS. In the DMS, 2 process were affected in acute females, 18 in acute males, 4 in protracted females, and 1 in protracted males. The top 10 biological pathways in the DMS are displayed in Table 9. Only 2 pathways were found to overlap between protracted females, and acute males and females (Figure 5H; Supplementary Table S3). The final assessment was done in the NAc, where we saw 17 enriched processes in acute females, 5 in acute male, 56 in protracted females, and 2 in protracted males. The top biological pathways in the NAc are displayed in Table 10. 2 pathways were found to overlap between protracted males and females, and 3 between protracted females and acute males (Figure 5I; Supplementary Table S3).

**TABLE 8:** Top 10 KEGG pathways in the DLS.

Description	Adjusted *p*-value	Description	Adjusted *p*-value
Protracted male		Protracted female	
Endocrine and other factor-regulated calcium reabsorption	4.53E-05	Pathways of neurodegeneration— multiple diseases	0.00059
Adrenergic signaling in cardiomyocytes	4.53E-05	Huntington disease	0.00059
Parkinson disease	1.30E-04	Carbon metabolism	0.00105
SNARE interactions in vesicular transport	1.30E-04	Parkinson disease	0.00105
Pathways of neurodegeneration—multiple diseases	1.32E-04	Amyotrophic lateral sclerosis	0.00105
Salivary secretion	1.65E-04	Oxidative phosphorylation	0.00166
Mineral absorption	1.69E-04	Metabolic pathways	0.00224
Mitophagy—animal	2.33E-04	Diabetic cardiomyopathy	0.00574
Amyotrophic lateral sclerosis	2.33E-04	Alzheimer disease	0.00704
Synaptic vesicle cycle	6.21E-04	Prion disease	0.00704
Acute female		Acute male	
Parkinson disease	2.10E-05	No significant pathways identified	
Pathways of neurodegeneration—multiple diseases	7.14E-05		
Huntington disease	1.00E-04		
Amyotrophic lateral sclerosis	1.11E-04		
Oxidative phosphorylation	1.23E-04		
Diabetic cardiomyopathy	5.40E-04		
Alzheimer disease	1.10E-03		
Metabolic pathways	1.16E-03		
Prion disease	1.54E-03		
Thermogenesis	1.54E-03		

Only KEGG pathways with an adjusted *p*-value < 0.05 are shown.

**TABLE 9:** Top 10 KEGG pathways in the DMS.

Description	Adjusted *p*-value	Description	Adjusted *p*-value
Protracted male		Protracted female	
Nucleocytoplasmic transport	0.0388	Ribosome	4.53E-09
		Coronavirus disease—COVID-19	1.44E-07
Acute male		Acute female	
Ribosome	4.39E-07	Ribosome	8.39E-10
Coronavirus disease—COVID-19	6.12E-06	Coronavirus disease—COVID-19	1.19E-06
Huntington disease	4.08E-04		
Endocrine and other factor-regulated calcium reabsorption	1.36E-03		
Glutamatergic synapse	1.47E-03		
Alcoholism	4.77E-03		
Systemic lupus erythematosus	6.90E-03		
Pathways of neurodegeneration—multiple diseases	1.01E-02		
Cocaine addiction	1.47E-02		
Synaptic vesicle cycle	1.68E-02		

Only KEGG pathways with an adjusted *p*-value < 0 .05 are shown.

**TABLE 10:** Top 10 KEGG pathways in the NAc.

Description	Adjusted *p*-value	Description	Adjusted *p*-value
Protracted male		Protracted female	
Regulation of actin cytoskeleton	0.0496	Non-alcoholic fatty liver disease	2.92E-05
Pathways of neurodegeneration - multiple diseases	0.0496	Thermogenesis	2.92E-05
		Oxidative phosphorylation	2.92E-05
		Parkinson disease	2.92E-05
		Amyotrophic lateral sclerosis	2.92E-05
		Prion disease	2.92E-05
		Ras signaling pathway	3.50E-05
		Retrograde endocannabinoid signaling	3.50E-05
		Pathways of neurodegeneration—multiple diseases	4.33E-05
		Diabetic cardiomyopathy	4.33E-05
Acute male		Acute female	
Ribosome	2.48E-11	No significant pathways identified	
Coronavirus disease—COVID-19	5.20E-08		
Spliceosome	1.90E-04		
Huntington disease	4.01E-02		

Only KEGG pathways with an adjusted *p*-value < 0.05 are shown.

## Discussion

Using this model of chronic intermittent two-bottle choice alcohol drinking in adult mice, we identified many differences in striatal protein abundance as a function of sex, duration of alcohol abstinence and striatal subregion. It is important to note that this model of drinking is not directly associated with producing alcohol dependence. We found that this model of alcohol drinking caused females to drink more alcohol than males, which is consistent with work from other studies, including our own (Hwa et al., 2011; Grecco et al., 2022). One limitation of our study is that we did not track blood alcohol levels. Others who have used a similar model have reported that our model produces BECs ranging from 79.58 to 166.93 mg/dL (Hwa et al., 2011). Though we did not find a sex effect on alcohol preference, female mice have been shown to display a greater preference for 20% alcohol than males with less sex differences at lower concentrations (Rivera-Irizarry et al., 2023). However, we have recently tested a model of binge-like alcohol drinking and found that males and females consumed similar levels of alcohol (Haggerty and Atwood, 2024). The differences in the sex effects between these studies may be a result of the type of access the mice had. Females also consumed more water than males, but the difference in amount was not as pronounced as seen with alcohol consumption. For this reason, we do not believe this difference in alcohol consumption was due to overall increased fluid consumption in females.

If we focus solely on the numbers of proteins with differential abundance because of alcohol drinking and different durations of abstinence, we find some interesting sex- and striatal subregion-dependent outcomes. In the DLS, females with acute abstinence and males with protracted abstinence displayed the greatest total changes in protein abundance. In contrast, in the DMS, acute abstinence produced more changes than protracted abstinence in both males and females. The total number of proteins affected were more modest than any of the changes seen in the DLS. In the NAc, both acute and protracted alcohol abstinence produced abundant changes in protein abundance in females, while protein abundance changes in males were largely restricted to acute abstinence. Taken together it appears that in males, acute abstinence produces the greatest changes in NAc and DMS, but over the course of protracted abstinence, protein abundance in these regions normalizes and changes in protein abundance shift to the DLS. In contrast, in females it appears that acute abstinence consistently produces protein abundance changes in all 3 striatal subregions, but the greatest change during protracted abstinence is in the NAc with very little effects in the DLS, which is the opposite of what is seen in males. Thus, the pattern of striatal protein abundance change in males and females appears to shift in spatially opposite directions. This is interesting as it has been reported that over the course of alcohol drinking, behavioral control of drinking shifts from ventromedial striatum to DLS (Willuhn et al., 2012; Bell et al., 2016). If we consider changes in protein abundance as a proxy for this control, then our data in males reflects this pattern, whereas in females it would be opposite. Of course, changes in protein abundance could just as well mediate a decrease in functional control as an increase. Much work will need to be done to link the changes in protein abundance seen here to changes in control over drinking behavior. We would also like to note that further work would be needed to isolate alterations that are due to alcohol intake versus differences in sensitivity to alcohol due to biological differences.

According to our hypothesis, we expected to see a significant change in the abundance of neuroinflammatory-related protein between drinking groups. Based on our prior work in adolescent rats, we specifically anticipated finding significant changes to the inflammatory-associated proteins endothelin-1 (ET-1), COX-2, and PGE_2_ in the dorsal striatum (Kline and Yamamoto, 2022). However, we did not identify significant alterations to these proteins in either of the DS subregions (DLS or DMS) even though we added probes for these to enhance detection during mass spectrometry. Not only did we not detect changes in the abundance of these proteins, our gene ontology and KEGG pathway assessments did not reveal a significant effect on any inflammation-related protein abundance. This was surprising to us. There are some potential explanations to account for this discrepancy. The most obvious difference is that we used mice here, whereas our previous work used rats (Kline and Yamamoto, 2022). Some studies show that there are distinct differences in components of the immune response between mice and rats, including variations in microglia function and chemokine release in neurons (Patrizio and Levi, 1994; Du et al., 2017; Lam et al., 2017). In addition, here the animals were adults when they started alcohol consumption, whereas our previous work used adolescents (Kline and Yamamoto, 2022). It is also possible that we did not identify the neuroinflammatory changes we expected to see due to the mice not consuming sufficient amounts, especially the males. Furthermore, several of the proteins had never been previously identified using mass spectrometry and are present at challenging to identify levels. Some proteins may also be changing in localization, splicing, or post translational modifications which we would not be able to identify in this study. Despite these species and age differences though, we still expected to see some changes in these inflammation pathways, even if it was not the same proteins that were affected. These discrepancies may be a product of the timing at which tissue samples were taken as our prior study did not find any differences in inflammation-related proteins early in abstinence, but these appeared over protracted abstinence. It is possible that in mice it takes longer for the abundance of these inflammation proteins to change.

Nevertheless, we conducted a PubMed database analysis of the overlapping proteins between protracted and acute abstinence males and females of each brain region (Figure 5) to identify any inflammatory-related role. From this search, we identified one neuroinflammatory-related protein that was significantly altered in both females with protracted abstinence and males with acute abstinence, Cluster of Differentiation CD200. CD200 is a key mediator of the innate immune response and neuroinflammation. It has also been shown to decrease in protein abundance in response to alcohol consumption in humans but increase during alcohol abstinence (Chaturvedi et al., 2020).

We investigated whether any of the proteins with significantly altered abundance were similarly altered in more than one group. We found very little overlap between all sex and abstinence groups within each striatal subregion. Focusing on similarities between sexes within each subregion, we found only 2 overlapping proteins in the protracted abstinence groups, but quite a few overlaps between sexes in the acute abstinence groups. These data suggest that alcohol drinking may produce some overlapping biological outcomes in both sexes, but over time these outcomes may diverge greatly. Nonetheless, in assessing what proteins are similarly affected in males and females, we identified some proteins relevant to alcohol. In the DLS, Fkbp5 was common to male and female mice that experienced acute abstinence. Fkbp5 is a glucocorticoid receptor binding protein that plays a strong role in psychiatric disorders and is induced by stress (Szymanska et al., 2009). The Fkbp5 gene can also be induced by alcohol as shown by its increased expression following acute alcohol injections (Kerns et al., 2005; Qiu et al., 2016). Furthermore, Fkbp5 knockout mice show increased alcohol consumption and blood alcohol levels compared to wildtype (Qiu et al., 2016). In addition to playing a role in alcohol consumption in mice, Fkbp5 was also identified as a potential key gene in human alcohol consumption. SUMO-conjugating enzyme UBC9 was common to male and female mice that underwent protracted abstinence as well as male drinkers that experienced acute abstinence. Similarly, UBC9 is tied to alcohol consumption as it plays a role in alcohol-induced liver damage (Tomasi et al., 2012; Tomasi et al., 2018). In the DMS, we identified proteasome 26S subunit, ATPase 1 (PSMC1), which was significantly altered in acute females and protracted males. PSMC1 displays decreased expression in liver tissue in response to alcohol treatment in mice. Decreased expression of PMSC1 is also identified in the livers of individuals who suffered from alcohol use disorder compared to healthy livers (Wang et al., 2016). Its role in the striatum is unknown. Our analysis of the NAc did not reveal any proteins that overlapped in drinking groups that were strongly linked to alcohol drinking. Additional study of the proteins we identified in these overlaps may produce new knowledge regarding the effects of alcohol on the brain that are common to both sexes.

We identified many biological process gene ontology terms that were affected by alcohol drinking and different lengths of abstinence. To aid in the interpretation of these data, we simplified our list based on term similarity using the Revigo online tool, to better elucidate the cellular functions and processes that were altered. This allowed us to cluster terms according to their cellular functions and their uniqueness (relative to the entire list of terms). We were able to do this for all groups except the DLS of males that underwent acute abstinence due to the limited number of significantly altered biological processes identified. As expected, brain development was one of the major clusters across all groups, which included processes such as neurogenesis, axonogenesis, neuron differentiation and many other neural development processes. Additionally, we were intrigued to see many processes related to synapse organization, chemical synaptic transmission, intracellular transport, and cytoskeleton organization that were common to all striatal subregions. In the DLS unique clusters included regulation of intracellular transduction, cell migration, cellular homeostasis, chemical synaptic transmission, and regulation of cellular component organization. In the DMS, unique clusters included mitochondrion localization, lipid modification and regulation of RNA export. Lastly, we saw major unique terms related to peptide metabolic processes, regulation of macromolecule localization, and apoptotic processes in the NAc.

A general feature across striatal subregions, sexes, and durations of alcohol abstinence are changes related to proteins that function to transport biological materials within cells, organization of cellular structure, and protein translation processes. These data would indicate that alcohol’s physiological and behavioral effects may be mediated by biochemical mechanisms that reorganize the structure and composition of striatal cells and in particular neurons. This is an important point to make as much current work in the field is focused on changes in mRNA transcription. Changes in the localization of proteins as well as in translation processes will likely not be detected using transcriptomic methodologies. For instance, the reduction of key organization proteins such as α- and β-tubulin and associated proteins is identified in brains of individuals with chronic alcohol consumption and rodents (Labisso et al., 2018). However, a slight increase was seen in tubulin gene expression. This further speaks to the relevance of assessing changes in translation due to the effects of alcohol. Furthermore, the trafficking of crucial components such as neurotransmitter receptors is modified by chronic alcohol consumption (Shen et al., 2011). In addition, studies of the structure of neurons will also be especially important. Indeed, studies show that alcohol-induced apoptosis disrupts neural plasticity (Toesca et al., 2003; Do et al., 2013). The effects on alcohol on neuronal developing is rather vast, and is known to also disrupt neurogenesis (Tateno and Saito, 2008).

Even though our gene ontology and pathway enrichment analyses did not find strong links to inflammation, some of our analyses pointed to changes in proteins related to neurodegenerative diseases, including Alzheimer disease, Huntington disease, amyotrophic lateral sclerosis, and Parkinson disease. We identified changes related to neurodegenerative diseases in the DLS and NAc, but not DMS, striatal subregions of both males and females that underwent protracted alcohol abstinence. We did not identify any link to these diseases in females with acute abstinence, but found some neurodegeneration-related changes in all striatal subregions of males with acute abstinence. These data would imply that alcohol consumption starts changes to protein pathways that lead to eventual neurodegeneration that begins early on in males that drink but develops more slowly in females over the course of a protracted abstinence period. Additional experimentation will be needed to see if this is a function of how much alcohol is consumed and for how long that consumption occurred over time. There has been an increased interest in the links between alcohol drinking and neurodegeneration in recent years (Kelso et al., 2011; Qin and Crews, 2012; Crews et al., 2015; Crews and Vetreno, 2016). While we did not find changes in the abundance of many specific inflammatory proteins, a key characteristic of many neurogenerative conditions is a neuroinflammatory response. Due to the strong relationship between these diseases and neuroinflammation, the neurogenerative pathways identified during this study may indicate that some sort of neurodegenerative inflammatory-related process may play a role in alcohol drinking and abstinence.

Overall, this study reveals many new candidate proteins to explore for how alcohol affects the brain to promote further alcohol use, and even possibly compulsive or habitual alcohol use. It reveals many differences between sexes as well as some overlapping targets between sexes. Further work is needed to determine why we observed changes in inflammation related protein abundances in prior work but failed to see similar outcomes here. However, our data implicate alcohol-induced changes in neurodegeneration-associated proteins and proteins involved in cellular transport, protein translation, and cellular structure that may also play a role in alcohol-induced deficits in brain health.

## Data Availability

The data presented in the study are deposited in a GitHub library at this link: https://github.com/bduffus/Chronic-Alcohol-Withdrawal-Proteome.
