# Supplementary figures and images for: The impact of abstinence from chronic alcohol consumption on the mouse striatal proteome: sex and subregion-specific differences
Source: Front Pharmacol. 2024 Jun 3;15:1405446. doi: 10.3389/fphar.2024.1405446 (PMC11180734; doi:10.3389/fphar.2024.1405446)

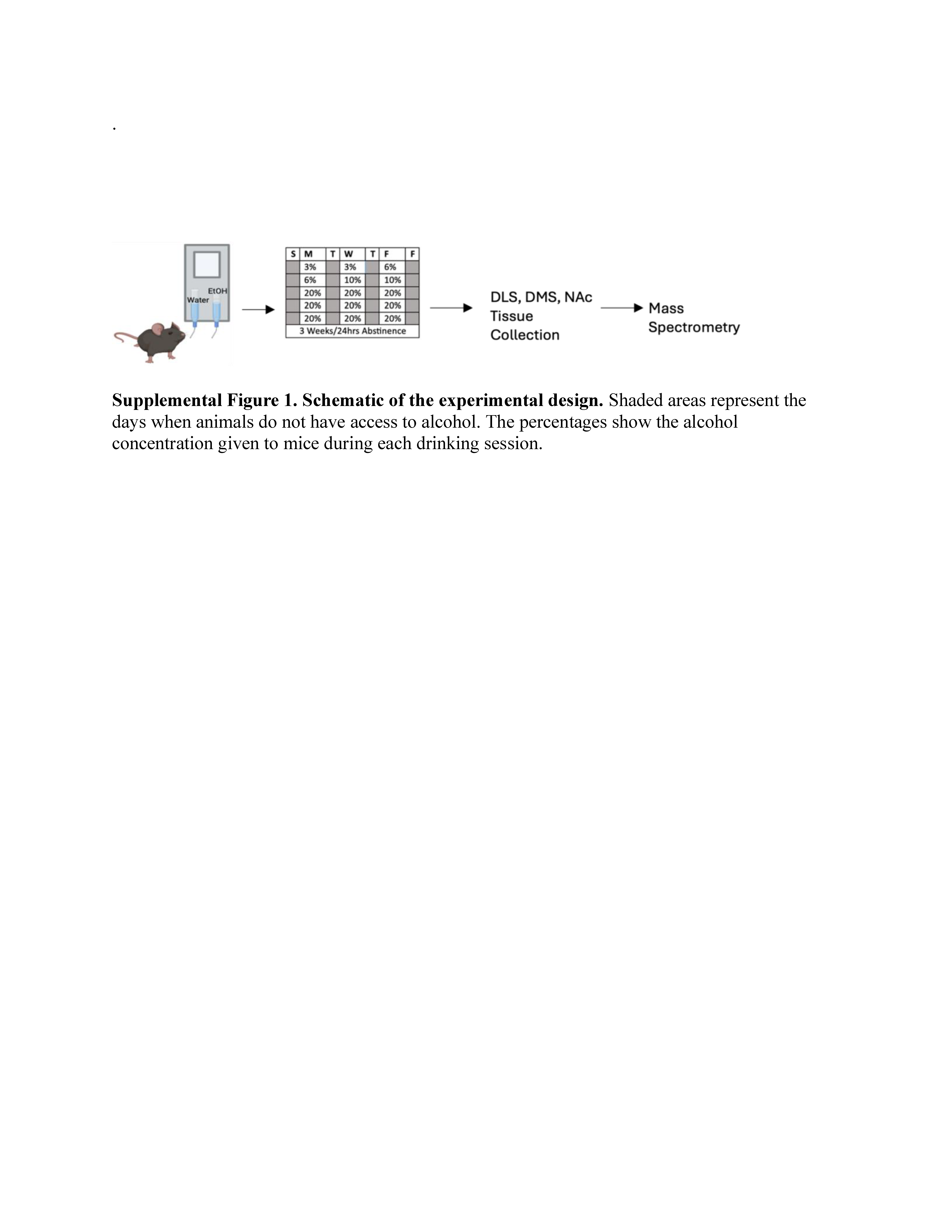

Supplement: Supplementary file 1 [file Image1.tiff]
