# Supplementary material for: The impact of abstinence from chronic alcohol consumption on the mouse striatal proteome: sex and subregion-specific differences
Source: Front Pharmacol. 2024 Jun 3;15:1405446. doi: 10.3389/fphar.2024.1405446 (PMC11180734; doi:10.3389/fphar.2024.1405446)
Supplement: Supplementary file 2 [file Table1.docx]

**Supplemental table 1.** Synthetic peptides used to create TMT trigger channels. Trigger channels were used to assist with the mass spectrometry quantification of specific proteins of interest.

| **Mouse Protein** | **Peptide (Thermo Fisher Scientific; >98% purity)** |
| --- | --- |
| Prostaglandin-endoperoxide synthase 2 (PTGS2; Q3UMR6) | ANPCCSNPCQNR |
|  | GLGHGVDLNHIYGETLDR |
|  | NVPIAVQAVAK |
|  | LDDINPTVLIK |
| Endothelin 1 receptor (EDNRA; Q61614) | IALSELHK |
|  | NQEQNNHNTER |
| Prostaglandin E Receptor 1 (PTGER1; P35375) | YELQYPGTWCFISLGPR |
|  | CVGVTQPLIHAAR |
