# Supplementary material for: The impact of abstinence from chronic alcohol consumption on the mouse striatal proteome: sex and subregion-specific differences
Source: Front Pharmacol. 2024 Jun 3;15:1405446. doi: 10.3389/fphar.2024.1405446 (PMC11180734; doi:10.3389/fphar.2024.1405446)
Supplement: Supplementary file 3 [file Table2.docx]

**Group names # total overlapping elements**

**DLS:** Acute M, Protract F, Protract M 1

positive regulation of protein metabolic process

**DLS:** Acute F, Protract F, Protract M 170

head development

regulation of locomotion

ribonucleoside triphosphate metabolic process

negative regulation of cellular component organization

energy derivation by oxidation of organic compounds

peptide biosynthetic process

supramolecular fiber organization

cell junction organization

cell morphogenesis

actin filament-based process

purine ribonucleotide biosynthetic process

regulation of postsynaptic membrane neurotransmitter receptor levels

protein localization to cell periphery

actin filament organization

regulation of transport

protein folding

regulation of cell motility

membrane organization

neuron differentiation

vesicle-mediated transport in synapse

protein localization to membrane

positive regulation of protein transport

neuron development

cell division

response to abiotic stimulus

nucleoside phosphate metabolic process

peptidyl-serine modification

cytoskeleton organization

small molecule metabolic process

cell adhesion

regulation of secretion

organophosphate metabolic process

inorganic cation transmembrane transport

establishment of protein localization

establishment or maintenance of cell polarity

negative regulation of protein metabolic process

organelle localization

positive regulation of transport

cell junction assembly

regulation of protein transport

nucleoside phosphate biosynthetic process

macromolecule catabolic process

plasma membrane bounded cell projection organization

cell part morphogenesis

generation of precursor metabolites and energy

cell morphogenesis involved in differentiation

regulation of vesicle-mediated transport

nucleoside triphosphate biosynthetic process

peptidyl-serine phosphorylation

ribonucleotide metabolic process

purine nucleoside triphosphate biosynthetic process

regulation of establishment of protein localization

plasma membrane bounded cell projection morphogenesis

locomotion

axon development

generation of neurons

cellular catabolic process

anterograde trans-synaptic signaling

cell projection organization

regulation of plasma membrane bounded cell projection organization

response to inorganic substance

ribonucleotide biosynthetic process

carbohydrate derivative biosynthetic process

postsynapse organization

cellular homeostasis

proton motive force-driven ATP synthesis

actin cytoskeleton organization

organic acid metabolic process

secretion

positive regulation of cellular component biogenesis

positive regulation of cell projection organization

modulation of chemical synaptic transmission

regulation of trans-synaptic signaling

purine ribonucleotide metabolic process

neuron projection development

ATP metabolic process

regulation of catalytic activity

nucleotide biosynthetic process

regulation of anatomical structure morphogenesis

ribose phosphate biosynthetic process

brain development

positive regulation of cellular component organization

organonitrogen compound biosynthetic process

synaptic signaling

signal release

cell-cell signaling

nucleobase-containing small molecule metabolic process

monoatomic cation transmembrane transport

nucleotide metabolic process

respiratory electron transport chain

regulation of protein localization to membrane

protein transport

translation

mitochondrion organization

regulation of secretion by cell

monoatomic ion transport

phosphorylation

cell motility

organonitrogen compound catabolic process

response to endogenous stimulus

cell projection morphogenesis

regulation of cell morphogenesis

synapse assembly

vesicle-mediated transport

regulation of cell projection organization

peptide metabolic process

secretion by cell

nucleoside triphosphate metabolic process

cell-substrate adhesion

purine-containing compound metabolic process

response to organonitrogen compound

protein localization to plasma membrane

purine nucleotide biosynthetic process

purine ribonucleoside triphosphate metabolic process

regulation of cellular component size

protein polymerization

mitochondrial respiratory chain complex assembly

chemical synaptic transmission

regulation of cell migration

regulation of protein localization

regulation of cellular localization

positive regulation of molecular function

intracellular transport

intracellular protein transport

cell migration

regulation of organelle organization

monoatomic ion transmembrane transport

cell-matrix adhesion

neuron projection morphogenesis

regulation of monoatomic ion transport

purine nucleoside triphosphate metabolic process

regulation of neuron projection development

cellular component disassembly

positive regulation of catalytic activity

oxoacid metabolic process

regulation of neurogenesis

aerobic respiration

cell morphogenesis involved in neuron differentiation

purine-containing compound biosynthetic process

carbohydrate derivative metabolic process

inorganic ion transmembrane transport

central nervous system development

ribonucleoside triphosphate biosynthetic process

amide metabolic process

purine ribonucleoside triphosphate biosynthetic process

regulation of protein modification process

carboxylic acid metabolic process

synapse organization

axonogenesis

neurogenesis

organophosphate biosynthetic process

ribose phosphate metabolic process

small molecule biosynthetic process

response to nitrogen compound

synaptic vesicle cycle

protein catabolic process

positive regulation of establishment of protein localization

purine nucleotide metabolic process

export from cell

oxidative phosphorylation

cellular component morphogenesis

transmembrane transport

electron transport chain

positive regulation of protein localization

protein-containing complex localization

trans-synaptic signaling

localization within membrane

regulation of anatomical structure size

cellular respiration

cellular response to oxygen-containing compound

**DLS:** Protract F, Protract M 92

regulation of metal ion transport

regulation of proteolysis involved in protein catabolic process

amino acid biosynthetic process

intracellular monoatomic cation homeostasis

intracellular chemical homeostasis

positive regulation of monoatomic ion transport

reactive nitrogen species metabolic process

negative regulation of transmembrane transport

fatty acid metabolic process

monoamine transport

regulation of ubiquitin-dependent protein catabolic process

cellular ketone metabolic process

glial cell differentiation

regulation of transmembrane transport

proteolysis involved in protein catabolic process

positive regulation of protein localization to membrane

chaperone-mediated protein complex assembly

modification-dependent protein catabolic process

regulation of intracellular protein transport

regulation of proteasomal ubiquitin-dependent protein catabolic process

positive regulation of transmembrane transport

regulation of kinase activity

cell projection assembly

actin filament bundle organization

carboxylic acid biosynthetic process

neuron cellular homeostasis

negative regulation of cell projection organization

protein phosphorylation

negative regulation of locomotion

monoatomic cation homeostasis

homeostatic process

positive regulation of DNA metabolic process

regulation of phosphorylation

proteasome-mediated ubiquitin-dependent protein catabolic process

protein localization to cell junction

intracellular calcium ion homeostasis

methylglyoxal metabolic process

positive regulation of multicellular organismal process

positive regulation of protein modification process

ubiquitin-dependent protein catabolic process

cell-cell adhesion

regulation of nitric oxide metabolic process

monocarboxylic acid metabolic process

gliogenesis

regulation of phosphate metabolic process

nitric oxide metabolic process

regulation of sodium ion transmembrane transport

regulation of sodium ion transport

negative regulation of cell motility

negative regulation of cell death

inorganic ion homeostasis

catecholamine transport

positive regulation of protein-containing complex assembly

regulation of protein phosphorylation

response to nutrient

positive regulation of protein binding

central nervous system neuron development

regulation of protein stability

regulation of transferase activity

protein stabilization

protein localization to organelle

response to organic cyclic compound

forebrain development

calcium ion homeostasis

positive regulation of intracellular transport

positive regulation of binding

regulation of protein catabolic process

monoatomic ion homeostasis

regulation of growth

positive regulation of developmental process

regulation of protein localization to cell periphery

proteasomal protein catabolic process

regulation of binding

regulation of monoatomic ion transmembrane transport

regulation of phosphorus metabolic process

regulation of protein localization to plasma membrane

regulation of catabolic process

regulation of cellular response to stress

regulation of proteasomal protein catabolic process

growth

regulation of protein binding

modification-dependent macromolecule catabolic process

chemical homeostasis

organic acid biosynthetic process

actin filament bundle assembly

positive regulation of intracellular protein transport

regulation of cell size

intracellular monoatomic ion homeostasis

proteolysis

plasma membrane bounded cell projection assembly

developmental growth

regulation of monoatomic cation transmembrane transport

**DLS:** Acute F, Protract M 121

establishment of protein localization to membrane

regulation of synapse structure or activity

actin filament polymerization

positive regulation of secretion by cell

response to oxidative stress

dendrite development

neuron migration

dendritic spine development

neuromuscular process

regulation of nervous system development

receptor-mediated endocytosis

cytokinesis

establishment of vesicle localization

behavior

regulation of hydrolase activity

NADH dehydrogenase complex assembly

regulation of GTPase activity

postsynaptic neurotransmitter receptor internalization

neurotransmitter secretion

developmental cell growth

regulation of receptor-mediated endocytosis

cognition

regulation of supramolecular fiber organization

positive regulation of supramolecular fiber organization

vesicle docking involved in exocytosis

learning or memory

amide transport

learning

locomotory behavior

vesicle docking

regulation of cytoskeleton organization

regulation of cellular component biogenesis

cell growth

organelle assembly

dephosphorylation

regulation of cell shape

nucleus localization

regulation of protein-containing complex assembly

postsynaptic density organization

regulation of cell death

negative regulation of signal transduction

axo-dendritic transport

positive regulation of cell growth

establishment of protein localization to mitochondrion

lipid metabolic process

phenol-containing compound metabolic process

cytoplasmic translation

Ras protein signal transduction

cell-substrate junction organization

regulation of intracellular signal transduction

neuron projection extension

regulation of actin filament length

negative regulation of response to stimulus

membrane docking

Golgi to plasma membrane transport

vesicle localization

inorganic anion transmembrane transport

synaptic vesicle endocytosis

cellular response to nitrogen compound

positive regulation of hydrolase activity

receptor internalization

nuclear migration

Golgi vesicle transport

amine transport

cytoskeleton-dependent intracellular transport

actin polymerization or depolymerization

calcium-ion regulated exocytosis

organelle localization by membrane tethering

ameboidal-type cell migration

exocytosis

exocytic process

endocytosis

dendrite morphogenesis

monoatomic cation transport

regulation of actin polymerization or depolymerization

organic hydroxy compound metabolic process

metal ion transport

synaptic vesicle exocytosis

endomembrane system organization

regulation of endocytosis

regulation of programmed cell death

regulation of actin cytoskeleton organization

postsynaptic specialization organization

microtubule-based process

positive regulation of nervous system development

positive regulation of locomotion

inner mitochondrial membrane organization

regulation of actin filament organization

regulated exocytosis

negative regulation of signaling

synaptic vesicle recycling

regulation of synapse organization

vesicle organization

vesicle-mediated transport to the plasma membrane

cellular component maintenance

mitochondrial transport

neurotransmitter transport

regulation of actin filament polymerization

regulation of neurotransmitter levels

small GTPase mediated signal transduction

developmental growth involved in morphogenesis

regulation of protein polymerization

regulation of cell junction assembly

postsynaptic endocytosis

mitochondrial respiratory chain complex I assembly

regulation of dendrite development

regulation of actin filament-based process

establishment of organelle localization

protein targeting

transport along microtubule

vesicle budding from membrane

positive regulation of GTPase activity

positive regulation of cell migration

response to insulin

signal release from synapse

microtubule-based transport

cellular response to organonitrogen compound

post-Golgi vesicle-mediated transport

negative regulation of cell communication

regulation of apoptotic process

negative regulation of supramolecular fiber organization

**DLS:** Acute F, Protract F 25

regulation of translation

cell cycle

cell cycle process

amide biosynthetic process

positive regulation of protein secretion

mitotic cell cycle

aerobic electron transport chain

postsynaptic cytoskeleton organization

response to acetylcholine

postsynaptic actin cytoskeleton organization

mitotic cell cycle process

ATP biosynthetic process

acetylcholine receptor signaling pathway

response to salt

cellular response to salt

positive regulation of developmental growth

hindbrain development

G protein-coupled acetylcholine receptor signaling pathway

carboxylic acid catabolic process

cell cycle phase transition

organic acid catabolic process

negative regulation of cell migration

response to metal ion

cellular response to acetylcholine

small molecule catabolic process

**Group names # total overlapping elements**

**DMS:** Acute F, Acute M, Protract F, Protract M 1

non-membrane-bounded organelle assembly

**DMS:** Acute F, Acute M, Protract M 23

negative regulation of cellular component organization

DNA metabolic process

dendrite development

cell cycle process

cytoskeleton organization

regulation of microtubule cytoskeleton organization

regulation of supramolecular fiber organization

regulation of DNA metabolic process

regulation of cytoskeleton organization

regulation of cellular component biogenesis

organelle assembly

regulation of organelle assembly

regulation of protein-containing complex assembly

mitotic cell cycle

mitotic cell cycle process

positive regulation of cellular component organization

regulation of organelle organization

protein localization to organelle

microtubule-based process

positive regulation of organelle organization

regulation of protein polymerization

microtubule cytoskeleton organization

neurogenesis

**DMS:** Acute F, Acute M, Protract F 26

peptide biosynthetic process

regulation of transport

ribosomal small subunit biogenesis

membrane organization

rRNA processing

amide biosynthetic process

ribosome biogenesis

establishment of protein localization

negative regulation of protein metabolic process

vesicle docking involved in exocytosis

ncRNA metabolic process

cytoplasmic translation

organonitrogen compound biosynthetic process

protein transport

translation

vesicle-mediated transport

peptide metabolic process

vesicle tethering involved in exocytosis

intracellular transport

intracellular protein transport

cellular component disassembly

translation at postsynapse

rRNA metabolic process

translation at presynapse

amide metabolic process

translation at synapse

**DMS:** Acute M, Protract M 14

regulation of microtubule polymerization

regulation of synaptic plasticity

positive regulation of cell cycle process

protein-DNA complex organization

chromatin remodeling

microtubule polymerization

postsynaptic density organization

chromatin organization

regulation of microtubule-based process

nucleosome organization

protein polymerization

postsynaptic specialization organization

growth

positive regulation of synaptic transmission

**DMS:** Acute F, Protract M 3

regulation of microtubule polymerization or depolymerization

chromosome organization

microtubule polymerization or depolymerization

**DMS:** Acute M, Protract F 6

vesicle-mediated transport in synapse

vesicle docking

regulation of mitochondrion organization

membrane docking

organelle localization by membrane tethering

endomembrane system organization

**DMS:** Acute F, Protract F 4

membrane fission

ncRNA processing

ribosomal large subunit biogenesis

organelle disassembly

**DMS:** Acute F, Acute M 150

lamellipodium organization

regulation of locomotion

maturation of SSU-rRNA

regulation of synapse structure or activity

supramolecular fiber organization

cellular response to peptide hormone stimulus

regulation of translation

response to oxidative stress

cell junction organization

response to xenobiotic stimulus

cell morphogenesis

cell cycle

actin filament-based process

dendritic spine development

actin filament organization

regulation of cell motility

cytoskeleton-dependent cytokinesis

neuron differentiation

protein localization to nucleus

protein localization to membrane

neuron development

regulation of GTPase activity

regulation of apoptotic signaling pathway

small molecule metabolic process

negative regulation of molecular function

organelle localization

regulation of transmembrane transport

positive regulation of transport

maintenance of protein localization in organelle

regulation of protein transport

positive regulation of protein depolymerization

ribosome assembly

positive regulation of protein metabolic process

macromolecule catabolic process

plasma membrane bounded cell projection organization

cell part morphogenesis

generation of precursor metabolites and energy

regulation of chromosome organization

regulation of vesicle-mediated transport

regulation of plasma membrane bounded cell projection assembly

positive regulation of programmed cell death

regulation of establishment of protein localization

plasma membrane bounded cell projection morphogenesis

locomotion

neuron cellular homeostasis

protein phosphorylation

generation of neurons

positive regulation of catabolic process

import into cell

cellular catabolic process

cell projection organization

regulation of plasma membrane bounded cell projection organization

positive regulation of DNA metabolic process

response to inorganic substance

postsynapse organization

cellular homeostasis

actin cytoskeleton organization

positive regulation of apoptotic process

organic acid metabolic process

cellular nitrogen compound catabolic process

positive regulation of cell projection organization

regulation of amide metabolic process

response to reactive oxygen species

neuron projection development

negative regulation of protein localization to nucleus

negative regulation of microtubule polymerization or depolymerization

regulation of catalytic activity

regulation of intracellular signal transduction

regulation of actin filament length

regulation of anatomical structure morphogenesis

response to toxic substance

synaptic signaling

cell-cell signaling

cellular response to nitrogen compound

mitochondrion organization

phosphorylation

cell motility

actin polymerization or depolymerization

positive regulation of signaling

cell projection morphogenesis

regulation of cell morphogenesis

regulation of cell projection organization

regulation of proteolysis

negative regulation of protein polymerization

exocytic process

endocytosis

dendrite morphogenesis

positive regulation of actin filament depolymerization

positive regulation of cell communication

positive regulation of plasma membrane bounded cell projection assembly

regulation of actin polymerization or depolymerization

regulation of cell migration

regulation of protein localization

regulation of cellular localization

regulation of transferase activity

regulation of endocytosis

dendritic spine morphogenesis

positive regulation of molecular function

regulation of programmed cell death

regulation of actin cytoskeleton organization

negative regulation of organelle organization

cell migration

response to organic cyclic compound

positive regulation of locomotion

negative regulation of cytoskeleton organization

neuron projection morphogenesis

regulation of actin filament organization

regulation of synapse organization

response to corticosteroid

mitotic cytokinesis

regulation of neuron projection development

ribosomal small subunit assembly

positive regulation of catalytic activity

regulation of actin filament polymerization

oxoacid metabolic process

cellular response to peptide

positive regulation of cell motility

small GTPase mediated signal transduction

regulation of dendrite morphogenesis

regulation of cell projection assembly

cell morphogenesis involved in neuron differentiation

positive regulation of lamellipodium organization

maintenance of protein location in cell

regulation of dendrite development

regulation of actin filament-based process

central nervous system development

establishment of organelle localization

lamellipodium assembly

negative regulation of protein-containing complex assembly

regulation of monoatomic ion transmembrane transport

vesicle targeting

regulation of protein modification process

carboxylic acid metabolic process

synapse organization

regulation of catabolic process

regulation of cellular response to stress

positive regulation of cell migration

regulation of protein binding

protein-containing complex disassembly

vesicle tethering

cellular response to organonitrogen compound

cellular component morphogenesis

transmembrane transport

positive regulation of protein localization

response to hydrogen peroxide

regulation of apoptotic process

negative regulation of supramolecular fiber organization

localization within membrane

regulation of anatomical structure size

cellular response to oxygen-containing compound

**Group names # total overlapping elements**

**NAc:** Acute F, Acute M, Protract F, Protract M 51

regulation of locomotion

ribonucleoside triphosphate metabolic process

regulation of synapse structure or activity

negative regulation of cellular component organization

cell junction organization

neuron projection organization

regulation of transport

vesicle-mediated transport in synapse

cytoskeleton organization

small molecule metabolic process

organophosphate metabolic process

positive regulation of protein metabolic process

macromolecule catabolic process

plasma membrane bounded cell projection organization

locomotion

import into cell

anterograde trans-synaptic signaling

cell projection organization

postsynapse organization

cytoplasmic translation

regulation of postsynapse organization

regulation of anatomical structure morphogenesis

organonitrogen compound biosynthetic process

synaptic signaling

receptor internalization

nucleotide metabolic process

cell motility

vesicle-mediated transport

peptide metabolic process

purine-containing compound metabolic process

purine ribonucleoside triphosphate metabolic process

regulation of cellular component size

chemical synaptic transmission

regulation of cell migration

regulation of protein localization

regulation of cellular localization

positive regulation of molecular function

intracellular transport

negative regulation of organelle organization

cell migration

regulation of organelle organization

microtubule-based process

regulation of synapse organization

purine nucleoside triphosphate metabolic process

cellular component disassembly

microtubule cytoskeleton organization

synapse organization

synaptic vesicle cycle

purine nucleotide metabolic process

oxidative phosphorylation

trans-synaptic signaling

**NAc:** Acute M, Protract F, Protract M 25

actin filament polymerization

cytosolic transport

supramolecular fiber organization

actin filament organization

regulation of supramolecular fiber organization

regulation of cytoskeleton organization

regulation of cellular component biogenesis

regulation of protein-containing complex assembly

actin filament bundle organization

positive regulation of protein modification process

regulation of actin filament length

actin polymerization or depolymerization

regulation of intracellular transport

exocytic process

protein polymerization

regulation of actin polymerization or depolymerization

negative regulation of cytoskeleton organization

dendritic spine organization

regulation of protein polymerization

positive regulation of developmental process

regulation of protein modification process

protein-containing complex disassembly

protein depolymerization

actin filament bundle assembly

negative regulation of supramolecular fiber organization

**NAc:** Acute F, Protract F, Protract M 15

actin filament-based process

proton motive force-driven mitochondrial ATP synthesis

neurotransmitter secretion

synaptic vesicle priming

nucleoside triphosphate biosynthetic process

purine nucleoside triphosphate biosynthetic process

ATP metabolic process

ATP biosynthetic process

organonitrogen compound catabolic process

synaptic vesicle exocytosis

neurotransmitter transport

ribonucleoside triphosphate biosynthetic process

purine ribonucleoside triphosphate biosynthetic process

signal release from synapse

protein catabolic process

**NAc:** Acute F, Acute M, Protract F 160

regulation of biological quality

energy derivation by oxidation of organic compounds

peptide biosynthetic process

presynapse organization

positive regulation of secretion by cell

amino acid metabolic process

positive regulation of metabolic process

cell morphogenesis

dendrite development

maintenance of postsynaptic specialization structure

establishment of localization in cell

dendritic spine development

synaptic vesicle recycling via endosome

regulation of cell motility

receptor-mediated endocytosis

membrane organization

neuron differentiation

regulation of biological process

protein localization to membrane

establishment of vesicle localization

amide biosynthetic process

neuron development

cellular component organization or biogenesis

nucleoside phosphate metabolic process

regulation of protein metabolic process

developmental process

positive regulation of macromolecule metabolic process

cell population proliferation

system development

macromolecule localization

establishment of protein localization

negative regulation of protein metabolic process

localization

multicellular organism development

organelle localization

regulation of transmembrane transport

positive regulation of transport

cellular macromolecule localization

synaptic vesicle localization

organelle organization

negative regulation of cellular process

cell junction assembly

regulation of protein transport

tRNA aminoacylation

endosomal transport

nervous system development

cell part morphogenesis

maintenance of synapse structure

generation of precursor metabolites and energy

cell development

cellular component biogenesis

regulation of vesicle-mediated transport

cellular metabolic process

establishment of localization

ribonucleotide metabolic process

regulation of localization

tRNA aminoacylation for protein translation

cellular process

postsynaptic density organization

regulation of establishment of protein localization

plasma membrane bounded cell projection morphogenesis

transport

presynaptic endocytosis

generation of neurons

organonitrogen compound metabolic process

regulation of response to stimulus

cellular catabolic process

regulation of plasma membrane bounded cell projection organization

phosphate-containing compound metabolic process

protein localization to cell junction

organic substance transport

cellular nitrogen compound biosynthetic process

secretion

primary metabolic process

modulation of chemical synaptic transmission

regulation of trans-synaptic signaling

purine ribonucleotide metabolic process

neuron projection development

Ras protein signal transduction

regulation of mitochondrion organization

negative regulation of catabolic process

regulation of cell communication

vesicle localization

positive regulation of cellular process

synaptic vesicle endocytosis

positive regulation of cellular component organization

cell-cell signaling

nucleobase-containing small molecule metabolic process

regulation of molecular function

protein transport

translation

mitochondrion organization

catabolic process

synaptic vesicle transport

protein localization to synapse

negative regulation of biological process

cell projection morphogenesis

regulation of cell morphogenesis

synapse assembly

protein localization

regulation of cell projection organization

cellular response to oxygen levels

carbohydrate catabolic process

regulation of protein-containing complex disassembly

protein metabolic process

secretion by cell

exocytosis

endocytosis

dendrite morphogenesis

regulation of cellular component organization

nitrogen compound transport

regulation of signaling

endomembrane system organization

regulation of endocytosis

metabolic process

intracellular protein transport

programmed cell death

protein localization to organelle

postsynaptic specialization organization

organic substance catabolic process

neuron projection morphogenesis

synaptic vesicle recycling

vesicle organization

regulation of neuron projection development

cellular component maintenance

regulation of cell population proliferation

mitochondrial transport

positive regulation of cellular metabolic process

cell junction maintenance

positive regulation of biological process

phosphorus metabolic process

cellular component organization

small GTPase mediated signal transduction

carbohydrate metabolic process

translation at postsynapse

aerobic respiration

positive regulation of endocytosis

cell morphogenesis involved in neuron differentiation

cellular response to stress

carbohydrate derivative metabolic process

establishment of organelle localization

translation at presynapse

amide metabolic process

biological regulation

vesicle budding from membrane

cellular component assembly

neurogenesis

regulation of cellular process

regulation of catabolic process

ribose phosphate metabolic process

amino acid activation

organic substance metabolic process

export from cell

positive regulation of nitrogen compound metabolic process

translation at synapse

cellular component morphogenesis

positive regulation of protein localization

cellular localization

localization within membrane

cellular respiration

**NAc:** Protract F, Protract M 30

positive regulation of transferase activity

filopodium assembly

endothelium development

cell adhesion

positive regulation of protein localization to membrane

cellular response to endogenous stimulus

regulation of kinase activity

proton motive force-driven ATP synthesis

actin cytoskeleton organization

regulation of protein tyrosine kinase activity

regulation of catalytic activity

regulation of intracellular signal transduction

cellular response to nitrogen compound

regulation of protein localization to membrane

response to endogenous stimulus

response to organonitrogen compound

regulation of transferase activity

regulation of actin cytoskeleton organization

regulation of actin filament organization

vesicle-mediated transport between endosomal compartments

regulation of actin filament polymerization

positive regulation of neuron apoptotic process

developmental growth involved in morphogenesis

regulation of protein localization to cell periphery

regulation of actin filament-based process

growth

response to nitrogen compound

cellular response to organonitrogen compound

regulation of anatomical structure size

developmental growth

**NAc:** Acute M, Protract M 5

negative regulation of actin filament polymerization

negative regulation of protein polymerization

viral translational termination-reinitiation

negative regulation of protein-containing complex assembly

negative regulation of dendritic spine maintenance

**NAc:** Acute F, Protract M 3

regulation of synaptic vesicle priming

negative regulation of response to stimulus

alcohol metabolic process

**NAc:** Acute M, Protract F 93

glucan metabolic process

response to oxidative stress

regulation of postsynaptic membrane neurotransmitter receptor levels

regulation of system process

rRNA processing

positive regulation of protein transport

positive regulation of phosphate metabolic process

cell differentiation

NADH dehydrogenase complex assembly

ribosome biogenesis

regulation of synaptic plasticity

negative regulation of transmembrane transport

modulation of excitatory postsynaptic potential

regulation of potassium ion transmembrane transport

Golgi organization

glycogen metabolic process

regulation of microtubule cytoskeleton organization

negative regulation of protein-containing complex disassembly

positive regulation of cell population proliferation

polysaccharide metabolic process

glycogen catabolic process

regulation of neuron apoptotic process

regulation of developmental process

negative regulation of protein depolymerization

negative regulation of monoatomic ion transmembrane transport

organelle assembly

regulation of cell shape

regulation of intracellular protein transport

cellular developmental process

negative regulation of cell projection organization

regulation of neurotransmitter receptor activity

vacuole organization

axo-dendritic transport

glucan catabolic process

energy reserve metabolic process

polysaccharide catabolic process

regulation of phosphorylation

establishment of protein localization to mitochondrion

cellular nitrogen compound catabolic process

protein targeting to mitochondrion

positive regulation of cellular component biogenesis

positive regulation of cell projection organization

heterocycle catabolic process

regulation of microtubule-based process

positive regulation of phosphorylation

negative regulation of potassium ion transmembrane transporter activity

mitochondrial membrane organization

regulation of phosphate metabolic process

anterograde axonal transport

actin filament depolymerization

ribosomal large subunit biogenesis

regulation of postsynaptic neurotransmitter receptor activity

regulation of signaling receptor activity

regulation of protein modification by small protein conjugation or removal

Golgi vesicle transport

protein localization to mitochondrion

phosphorylation

regulation of synapse assembly

negative regulation of potassium ion transport

neuron apoptotic process

organelle disassembly

negative regulation of cation transmembrane transport

organic cyclic compound catabolic process

axonal transport

mitochondrial respiratory chain complex assembly

protein-containing complex organization

positive regulation of phosphorus metabolic process

positive regulation of nervous system development

protein-containing complex assembly

inner mitochondrial membrane organization

negative regulation of potassium ion transmembrane transport

positive regulation of organelle organization

positive regulation of intracellular transport

negative regulation of actin filament depolymerization

regulation of protein depolymerization

regulation of cell junction assembly

mitochondrial respiratory chain complex I assembly

presynapse assembly

regulation of binding

endoplasmic reticulum to Golgi vesicle-mediated transport

protein targeting

regulation of actin filament depolymerization

regulation of phosphorus metabolic process

nucleobase-containing compound catabolic process

regulation of small molecule metabolic process

positive regulation of establishment of protein localization

aromatic compound catabolic process

regulation of potassium ion transport

positive regulation of intracellular protein transport

neurotransmitter receptor internalization

circulatory system process

protein-containing complex localization

regulation of nervous system process

**NAc:** Acute F, Protract F 133

regulation of metal ion transport

regulation of proteolysis involved in protein catabolic process

regulation of neuronal synaptic plasticity

regulation of transporter activity

alpha-amino acid metabolic process

peptide secretion

establishment of protein localization to extracellular region

purine ribonucleotide biosynthetic process

presynaptic dense core vesicle exocytosis

protein localization to cell periphery

positive regulation of insulin secretion

behavior

response to stimulus

protein localization to extracellular region

regulation of signal transduction

protein secretion

negative regulation of molecular function

regulation of secretion

inorganic cation transmembrane transport

regulation of ubiquitin-dependent protein catabolic process

nucleoside monophosphate metabolic process

regulation of peptide hormone secretion

proteolysis involved in protein catabolic process

monosaccharide catabolic process

regulation of amyloid precursor protein catabolic process

amide transport

locomotory behavior

negative regulation of proteolysis involved in protein catabolic process

regulation of monoatomic ion transmembrane transporter activity

regulation of regulated secretory pathway

response to endoplasmic reticulum stress

nucleoside phosphate biosynthetic process

receptor localization to synapse

positive regulation of peptide secretion

modification-dependent protein catabolic process

epithelial cell development

regulation of proteasomal ubiquitin-dependent protein catabolic process

positive regulation of protein secretion

positive regulation of programmed cell death

insulin secretion

negative regulation of proteasomal protein catabolic process

ATP synthesis coupled electron transport

axon development

hormone secretion

neuron cellular homeostasis

amino acid transport

cell death

regulation of peptide transport

ribonucleotide biosynthetic process

proteasome-mediated ubiquitin-dependent protein catabolic process

cellular homeostasis

aerobic electron transport chain

regulation of neurotransmitter secretion

positive regulation of apoptotic process

organic acid metabolic process

ubiquitin-dependent protein catabolic process

regulation of hormone secretion

cellular response to hypoxia

positive regulation of peptide hormone secretion

regulation of transmembrane transporter activity

nucleotide biosynthetic process

positive regulation of hormone secretion

ribose phosphate biosynthetic process

brain development

signal release

anterograde axonal protein transport

monoatomic cation transmembrane transport

respiratory electron transport chain

protein localization to presynapse

cytoskeleton-dependent intracellular transport

regulation of secretion by cell

regulation of synaptic vesicle exocytosis

monoatomic ion transport

regulation of protein secretion

positive regulation of secretion

positive reg. of aspartic-type endopeptidase activity in amyloid precursor protein catabolic process

nucleoside triphosphate metabolic process

intrinsic apoptotic signaling pathway

regulation of proteolysis

process utilizing autophagic mechanism

hormone transport

protein localization to plasma membrane

purine nucleotide biosynthetic process

intracellular signal transduction

regulation of peptide secretion

endothelial cell development

carboxylic acid catabolic process

apoptotic process

monoatomic cation transport

purine nucleoside monophosphate metabolic process

positive regulation of aspartic-type peptidase activity

anatomical structure development

regulation of programmed cell death

negative regulation of protein localization

autophagy

monoatomic ion transmembrane transport

organic acid catabolic process

regulated exocytosis

mitochondrial electron transport, NADH to ubiquinone

peptide hormone secretion

regulation of monoatomic ion transport

peptide transport

regulation of protein catabolic process

oxoacid metabolic process

negative regulation of ubiquitin-dependent protein catabolic process

regulation of neurotransmitter levels

pallium development

signaling

purine-containing compound biosynthetic process

inorganic ion transmembrane transport

regulation of insulin secretion

cellular response to decreased oxygen levels

proteasomal protein catabolic process

regulation of neurotransmitter transport

regulation of monoatomic ion transmembrane transport

mitochondrial ATP synthesis coupled electron transport

transport along microtubule

establishment of endothelial barrier

carboxylic acid metabolic process

cell communication

axonogenesis

organophosphate biosynthetic process

small molecule biosynthetic process

regulation of proteasomal protein catabolic process

apoptotic signaling pathway

modification-dependent macromolecule catabolic process

microtubule-based transport

proteolysis

electron transport chain

regulation of membrane potential

regulation of apoptotic process

small molecule catabolic process

regulation of monoatomic cation transmembrane transport

**NAc:** Acute F, Acute M 27

synaptic vesicle budding from presynaptic endocytic zone membrane

regulation of synaptic vesicle recycling

synaptic vesicle clustering

citrate metabolic process

viral translation

protein folding

response to abiotic stimulus

regulation of dendritic spine morphogenesis

release of cytochrome c from mitochondria

positive regulation of amide metabolic process

positive regulation of synaptic vesicle clustering

positive regulation of neuron projection development

protein modification process

positive regulation of synaptic vesicle recycling

regulation of amide metabolic process

apoptotic mitochondrial changes

nitrogen compound metabolic process

macromolecule modification

peptidyl-amino acid modification

regulation of synaptic vesicle endocytosis

dendritic spine morphogenesis

response to stress

regulation of dendrite development

regulation of dendritic spine development

positive regulation of dendritic spine development

mitochondrial electron transport, cytochrome c to oxygen

positive regulation of synaptic vesicle endocytosis
