# Supplementary material for: The impact of abstinence from chronic alcohol consumption on the mouse striatal proteome: sex and subregion-specific differences
Source: Front Pharmacol. 2024 Jun 3;15:1405446. doi: 10.3389/fphar.2024.1405446 (PMC11180734; doi:10.3389/fphar.2024.1405446)
Supplement: Supplementary file 4 [file Table3.docx]

| **DLS** | |
| --- | --- |
| **Acute F vs Protracted F vs Protracted Male** | **Acute**  **F vs. Protracted F** |
| Amyotrophic lateral sclerosis | Diabetic cardiomyopathy |
| Parkinson disease | Metabolic pathways |
| Huntington disease | Oxidative phosphorylation |
| Pathways of neurodegeneration - multiple diseases |  |
| Prion disease |  |
| Alzheimer disease |  |
| **DMS** | |
| **Acute F vs Acute M vs Protracted F** |  |
| Ribosome |  |
| Nucleocytoplasmic transport |  |
| **NAc** | |
| **Protracted F vs Protracted M** | **Acute M vs Protracted F** |
| Pathways of neurodegeneration - multiple diseases | Coronavirus disease - COVID-19 |
| Regulation of actin cytoskeleton | Ribosome |
|  | Huntington disease |

**Supplemental table 3. Overlapping biological pathways in striatal subregions.**
